# Pharmacological SERCA activation limits diet-induced steatohepatitis and restores liver metabolic function in mice

**DOI:** 10.1016/j.jlr.2024.100558

**Published:** 2024-05-08

**Authors:** Tomasz K. Bednarski, Mohsin Rahim, Clinton M. Hasenour, Deveena R. Banerjee, Irina A. Trenary, David H. Wasserman, Jamey D. Young

**Affiliations:** 1 Department of Chemical and Biomolecular Engineering, Vanderbilt University, Nashville, TN, USA; 2 Department of Molecular Physiology and Biophysics, Vanderbilt University, Nashville, TN, USA

**Keywords:** hepatology, nonalcoholic fatty liver disease, metabolic dysfunction-associated steatotic liver disease, nonalcoholic steatohepatitis, metabolic dysfunction-associated steatohepatitis, metabolism, metabolic flux analysis, citric acid cycle, pyruvate cycling, polyunsaturated fatty acids, calcium, sarcoplasmic/endoplasmic reticulum calcium-transporting ATPase

## Abstract

Metabolic dysfunction-associated steatotic liver disease is the most common form of liver disease and poses significant health risks to patients who progress to metabolic dysfunction-associated steatohepatitis. Fatty acid overload alters endoplasmic reticulum (ER) calcium stores and induces mitochondrial oxidative stress in hepatocytes, leading to hepatocellular inflammation and apoptosis. Obese mice have impaired liver sarco/ER Ca^2+^-ATPase (SERCA) function, which normally maintains intracellular calcium homeostasis by transporting Ca^2+^ ions from the cytoplasm to the ER. We hypothesized that restoration of SERCA activity would improve diet-induced steatohepatitis in mice by limiting ER stress and mitochondrial dysfunction. WT and melanocortin-4 receptor KO (*Mc4r*^−/−^) mice were placed on either chow or Western diet (WD) for 8 weeks. Half of the WD-fed mice were administered CDN1163 to activate SERCA, which reduced liver fibrosis and inflammation. SERCA activation also restored glucose tolerance and insulin sensitivity, improved histological markers of metabolic dysfunction-associated steatohepatitis, increased expression of antioxidant enzymes, and decreased expression of oxidative stress and ER stress genes. CDN1163 decreased hepatic citric acid cycle flux and liver pyruvate cycling, enhanced expression of mitochondrial respiratory genes, and shifted hepatocellular [NADH]/[NAD^+^] and [NADPH]/[NADP^+^] ratios to a less oxidized state, which was associated with elevated PUFA content of liver lipids. In sum, the data demonstrate that pharmacological SERCA activation limits metabolic dysfunction-associated steatotic liver disease progression and prevents metabolic dysfunction induced by WD feeding in mice.

## Graphical abstract

Key effects of CDN1163 on hepatic steatosis phenotypes in Western diet-fed mice. Activation of sarco/endoplasmic reticulum Ca^2+^-ATPase (SERCA) reduced hepatic endoplasmic reticulum stress and oxidative stress while improving mitochondrial function and shifting hepatocellular redox to a less oxidized state. SERCA activation also reduced body weight and fat mass of Western diet-fed mice, possibly due to actions of CDN1163 in peripheral tissues that result in increased whole-body energy expenditure. The combined hepatic and extrahepatic effects of CDN1163 contributed to restoring PUFA content of liver lipids and inhibiting inflammation and fibrosis associated with the progression from metabolic dysfunction-associated steatotic liver disease to metabolic dysfunction-associated steatohepatitis.

**Figure:**
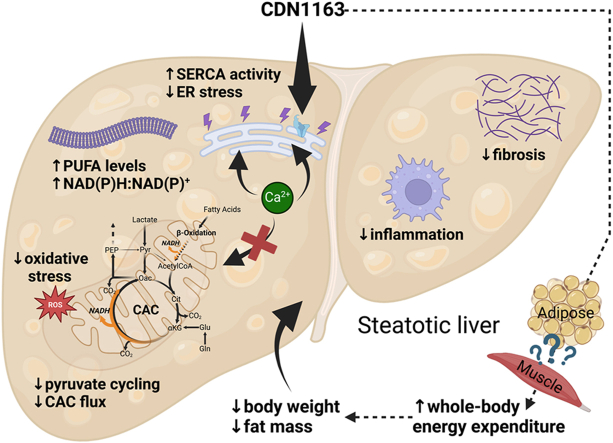

Metabolic dysfunction-associated steatotic liver disease (MASLD) is the hepatic manifestation of metabolic syndrome and the most common form of liver disease, affecting nearly 30% of the US population. Chronic inflammation and progressive fibrosis caused by prolonged MASLD may lead to metabolic dysfunction-associated steatohepatitis (MASH) and later to cirrhosis and hepatocellular carcinoma. Pharmacological treatments that prevent progression from MASLD to MASH have yet to be approved by the Food and Drug Administration. Therefore, better understanding of both metabolic and molecular mechanisms of MASH pathogenesis is needed to develop new strategies for prevention and effective treatment of MASLD and related disorders.

Growing evidence points toward mitochondrial dysfunction as a focal point in MASH and hepatocellular carcinoma pathogenesis by affecting hepatocyte bioenergetics, reactive oxygen species (ROS) homeostasis, endoplasmic reticulum (ER) stress, inflammation, and cell death (1). While short-term elevations in mitochondrial metabolism may provide an adaptive mechanism to dispose of excess liver fat, chronic hyperactivation of oxidative metabolic pathways leads to gradual loss of mitochondrial function and hepatocellular damage in MASH patients (2). We have previously shown that exogenous fatty acids (FAs), particularly long-chain saturated FAs (SFAs), can alter mitochondrial metabolism independently of β-oxidation in vitro. Palmitate overload promotes Ca^2+^ leakage from ER to mitochondria, leading to hyperactivation of citric acid cycle (CAC) enzymes and anaplerotic fluxes, increased cellular respiration, and accumulation of ROS that triggers cell death in cultured hepatocytes (3, 4, 5).

Ca^2+^ ions are potent intracellular secondary messengers that regulate a variety of crucial metabolic and signaling pathways. Sarco/endoplasmic reticulum Ca^2+^-ATPase (SERCA) is the main regulator of intracellular Ca^2+^ and actively pumps calcium into the ER where it is stored, thereby maintaining cytosolic Ca^2+^ homeostasis. However, changes in ER lipid composition are accompanied by a disturbance of Ca^2+^ homeostasis and Ca^2+^ leakage at ER–mitochondria contact sites (6). SERCA protein and mRNA levels are dramatically reduced in the livers of obese mice compared to lean animals, which significantly impairs Ca^2+^ transport from the cytosol to the ER (7) and promotes hepatic ER stress in vivo (8). It has been therefore suggested that restoration of Ca^2+^ homeostasis and subsequent mitigation of ER stress might be a promising treatment to reduce hepatocellular lipotoxicity and limit MASLD progression (9).

Recently, the small molecule allosteric activator CDN1163 was found to dose dependently increase the *V*_*max*_ of SERCA isoforms found in the liver, heart, and skeletal muscle (10, 11). Kang *et al.* (10) showed that CDN1163 increases SERCA activity and improves liver Ca^2+^ transport in ER microsomes isolated from mouse liver tissue. In genetically obese *ob/ob* mice, acute CDN1163 treatments (50 mg/kg for five consecutive days) reduced fasting glucose, decreased adipose tissue weight, ameliorated liver steatosis, and increased energy expenditure without impacting food intake or total body weight. However, the effects of prolonged CDN1163 administration to limit the progression from MASLD to MASH have not been studied, and the impact of CDN1163 treatments on in vivo metabolic fluxes are unknown. In this study, we hypothesized that CDN1163 would protect obese mice from liver inflammation and fibrosis during chronic high-fat feeding by limiting ER stress and mitochondrial dysfunction.

Our hypothesis was tested by examining the impact of CDN1163 on MASLD markers and metabolic phenotypes of wild type (WT) and melanocortin-4 receptor deficient (*Mcr4*^−/−^, KO) mice. Mice with disrupted melanocortin-4 hypothalamic signaling exhibit chronic hyperphagia and develop human-like MASH symptoms upon Western diet (WD) feeding (12, 13). Age-matched WT and KO mice were maintained on chow or WD for 8 weeks to establish varying levels of MASLD severity. Additional groups of mice were administered regular CDN1163 injections throughout the entire 8-week study period. Mice were examined for changes in their whole-body metabolic phenotype, liver and plasma lipid profiles, and markers of liver inflammation and fibrosis. Stable and radioactive isotopes were used to assess in vivo metabolic fluxes, and targeted metabolomics was applied to profile changes in key intermediary metabolites and liver redox ratios. Finally, changes in expression of genes involved in FA metabolism and intracellular stress signaling pathways were assessed to provide mechanistic insight to the effects of CDN1163.

## Materials and methods

### Animals and diets

**Fig. 1:**
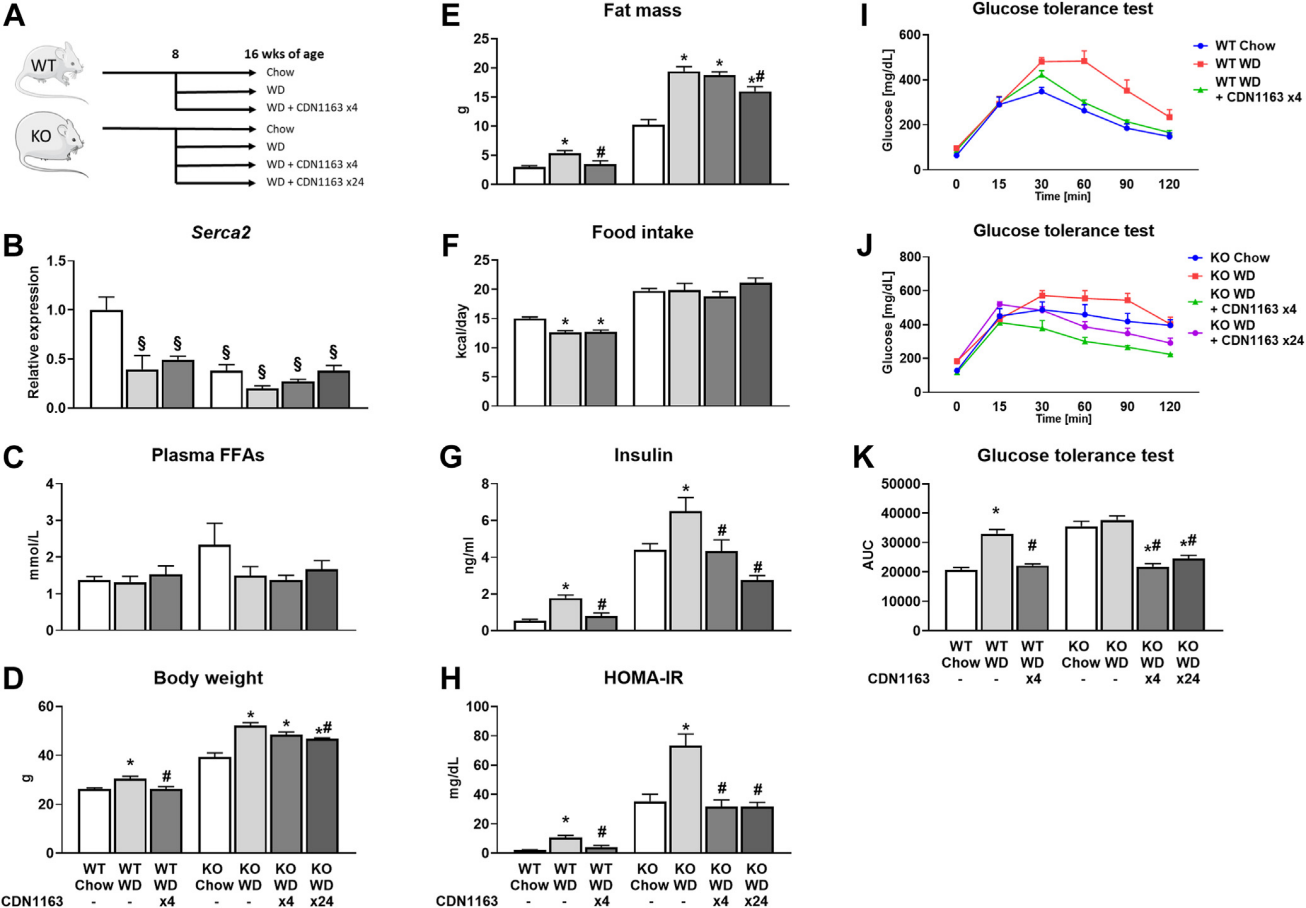
Metabolic response to WD feeding and CDN1163 treatment. Diagram of overall study design indicating experimental groups (A). After weaning, mice were placed on a standard chow diet. At 8 weeks of age, WT or *Mc4r*^*−/−*^ (KO) mice were either maintained on chow or switched to WD (±CDN1163) for another 8 weeks. One cohort of WT mice and one cohort of KO WD-fed mice were given CDN1163 injections once every 2 weeks (four in total) and one cohort of KO WD-fed mice was given CDN1163 injections three times a week (24 in total). All mice were 16 weeks of age upon completion of the study. mRNA expression of *Serca2* (B). Data are presented as mean ± SEM (n ≥ 6). §*P* < 0.05 versus WT chow. Measurements of plasma free fatty acid levels (C), body weight (D), whole-body fat mass (E), average daily food intake (F), plasma insulin level (G), HOMA-IR (H), and glucose tolerance of WT (I) and KO (J) mice. Area under the curve (AUC) calculated from glucose tolerance test results (K). Data are presented as mean ± SEM (n ≥ 8). ∗*P* < 0.05 versus chow, #*P* < 0.05 versus WD. HOMA-IR, homeostatic model assessment for insulin resistance; SERCA, sarco/endoplasmic reticulum Ca^2+^-ATPase; WD, Western diet.

All in vivo procedures were performed with approval from the Vanderbilt Institutional Animal Care and Use Committee. Experiments were performed on 16-week-old male C57Bl/6J (WT) mice or *Mc4r*^*−/−*^ (KO) mice on the same genetic background. Animals were housed in a humidity and temperature (23°C) stable environment and maintained on a 12:12 h light/dark cycle. Mice were given ad libitum access to food and water. After weaning, mice were provided a standard chow diet (5L0D, 30% protein, 57% carbohydrates, 13% fat by caloric contribution; LabDiet, St. Louis, MO). At 8 weeks of age, some randomly selected mice were switched to WD that is high in sucrose, cholesterol, and saturated fat (D12079B, 17% protein, 43% carbohydrates, and 40% fat by caloric contribution; Research Diets Inc, New Brunswick, NJ) for another 8 weeks. Two groups of WD-fed animals (WT and KO) were given CDN1163 injections (50 mg/kg body weight) once every 2 weeks for the entire 8-week diet period (four total doses). Because some effects of CDN1163 were less pronounced in KO mice than WT, an additional group of WD-fed KO animals was administered CDN1163 at the same dose but at a higher frequency of three times per week during the 8-week diet period (24 total doses). All remaining animals were administered vehicle (10% DMSO and 10% Tween 80 in 0.9% NaCl) using identical regimens. Seven groups were studied in total: WT chow, WT WD, WT WD +CDN1163 x4, KO chow, KO WD, KO WD +CDN1163 x4, and KO WD +CDN1163 x24 (n ≥ 6) (Fig. 1A). The group size was based on a previous, similar study by Hasenour *et al.* (14).

### In vivo procedures

Glucose tolerance tests were performed ∼1 week prior to the end of the study, as described previously (15). Approximately 5 days prior to the end of the study, ∼50% of the mice from each cohort had catheters surgically implanted in the jugular vein and carotid artery for infusing and sampling, respectively, as previously described (16). The other half did not undergo surgery, but otherwise were maintained similarly. In vivo tracer infusions were performed as described in detail elsewhere (17) with modifications described in Supplemental Methods. Plasma samples and tissues were harvested and stored at −80°C prior to analysis.

### ‬‬‬‬‬‬‬‬‬‬‬‬‬‬‬‬‬‬‬‬‬‬‬‬‬‬‬‬‬‬‬‬‬‬‬‬‬‬‬‬‬‬‬‬‬‬‬‬‬‬‬‬‬‬‬‬‬‬‬‬‬‬‬‬‬‬‬‬‬‬‬‬‬‬‬‬‬‬‬‬‬‬‬‬‬Liver metabolic flux analysis

Following protein precipitation with cold acetone, plasma samples were derivatized as described in detail elsewhere (18). Glucose and other metabolite derivatives were analyzed by GC-MS as described previously (19). Metabolites were identified through comparison to a library of known standards, and the accuracy of mass isotopomer distribution measurements were validated by measurement of unenriched control samples. An expanded, two-compartment metabolic model of liver and extrahepatic metabolism (Table S1) was constructed using the INCA software tool (20) (accessible at http://mfa.vueinnovations.com/mfa) as described previously (17). Further details are provided in Supplemental Methods.

### Liver histology

Liver tissues were fixed in 10% neutral buffered formalin, routinely processed and embedded in paraffin, and cut into 5 μm sections. H&E stained sections of liver from each mouse were evaluated for evidence of MASLD by a board-certified veterinary pathologist in blinded fashion. Scoring of MASH was based on previously published criteria (21). Further details are provided in Supplemental Methods.

### Plasma analyses, body composition, and liver lipids measurements

Body composition was assessed using a Bruker Minispec benchtop pulsed NMR (7 T) system (model mq7.5) (Bruker, Billerica, MA). Insulin was measured with radioimmunoassay RI-13K (MilliporeSigma, Burlington, MA). Plasma free fatty acids (FFAs) were evaluated using the HR Series NEFA-HR(2) kit (FUJIFILM Wako Diagnostics USA Corporation, Richmond, VA). Following Folch’s extraction, liver lipids were separated using thin layer chromatography and analyzed by GC, as described in the Supplemental Methods.

### Quantification of tissue and plasma metabolites

Plasma and tissue metabolites from catheterized mice were measured using a standard curve method. Absolute quantification of metabolites was performed by running calibration standards along with extracted samples. Plasma and tissue metabolites were normalized to the plasma volume and tissue weight, respectively. Cytosolic and mitochondrial redox states for liver and plasma were estimated using enzymatic equilibrium relations described elsewhere (22) and further detailed in the Supplemental Methods.

### Analysis of mRNA expression

RNA was isolated from ∼20 mg of liver using the RNeasy Mini Kit (Qiagen, Hilden, Germany). Liver gene expression was assessed using either quantitative real-time PCR for targeted mRNA transcripts or the NanoString murine nCounter Metabolic Pathways Panel (NanoString Technologies, Seattle, WA) for global analysis of mRNA expression and pathway enrichment. Further details are provided in Supplemental Methods.

### Statistics

Unless otherwise specified, data are presented as means ± SEM. GraphPad Prism software (www.graphpad.com) was used for statistical analysis. Differences between the groups were tested using ANOVA and Tukey multiple comparisons post hoc analysis. Analysis of histological scoring was performed using Kruskal-Wallis ANOVA with a Dunn’s multiple comparisons test for post hoc analysis. Significant differences were defined as *P* < 0.05.

## Results

### SERCA activation improves metabolic phenotypes of obese mice in a dose-dependent manner

WD feeding significantly increased body weight and fat mass (Fig. 1D, E), increased fasting plasma insulin (Fig. 1G) and homeostatic model assessment for insulin resistance score (Fig. 1H), and reduced glucose tolerance (Fig. 1I–K) compared to control, chow-fed animals. Consistent with a previous report (8), *Serca2* expression in liver was decreased in WD-fed animals (Fig. 1B). Expression levels of the calcium-binding proteins S100 calcium-binding protein A1 (*S100A1*) and regucalcin (*Rgn*), which correlate with SERCA activity (23, 24), were similarly decreased by WD feeding in WT (*S100A1* only) and KO mice (supplemental Fig. S1A, B). Treating WT mice with CDN1163 once every 2 weeks (x4) rescued *S100A1* expression and elevated *Rgn* expression, indicative of liver SERCA activation. Similar effects were observed in KO mice with increased CDN1163 dosing frequency (three times a week, x24) (supplemental Fig. S1A, B). These dosing regimens significantly decreased body weight and fat mass of WD-fed mice of both genotypes (Fig. 1D, E). Changes in body weight and fat mass were not correlated with food intake (Fig. 1F), suggesting that energy expenditure was elevated in CDN1163-treated animals. While plasma FFA levels were not different among the groups (Fig. 1C), CDN1163 significantly decreased fasting plasma insulin levels (Fig. 1G) and restored glucose tolerance (Fig. 1I–K) and homeostatic model assessment for insulin resistance score (Fig. 1H) to control levels. In summary, SERCA activation by CDN1163 treatment improved the metabolic phenotypes of obese mice and produced dose-dependent changes in the KO WD-fed cohort. Since the effects of CDN1163 on KO mice were less pronounced with biweekly dosing, the KO WD +CDN1163 x4 group was omitted from subsequent analyses.

### SERCA activation reduces hepatic fibrosis and histological markers of MASH in WD-fed mice

**Fig. 2:**
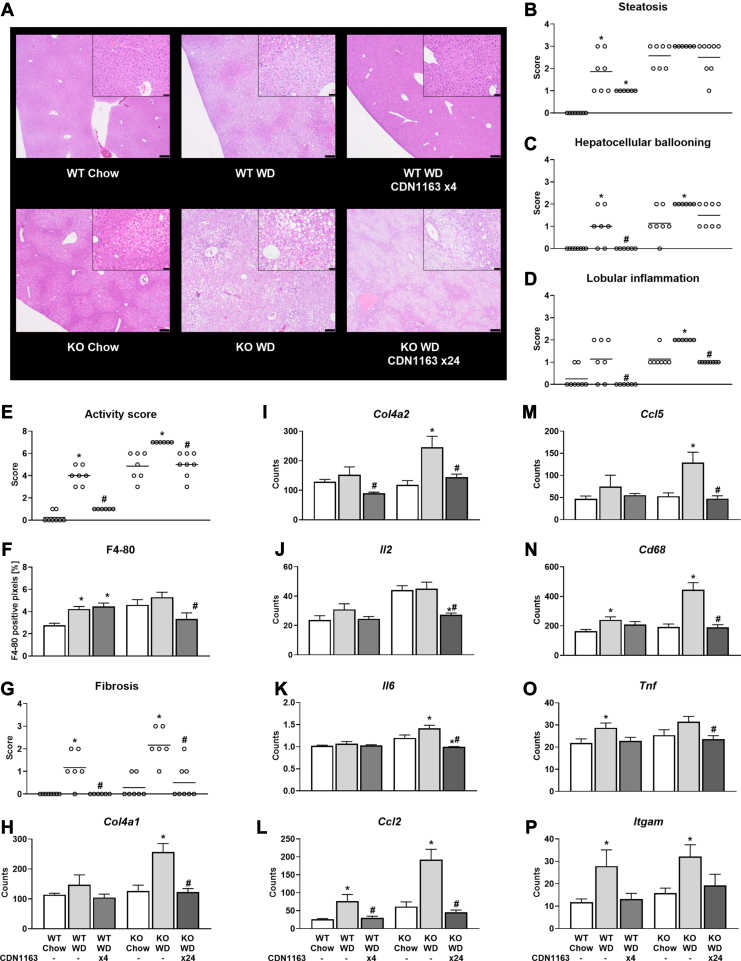
Liver histology and expression of genes involved in fibrogenesis and inflammation. Liver sections from 16-week-old mice stained with H&E (A). Scale bar = 200 μm (inset scale bar = 50 μm). Scoring of steatosis (B), hepatocellular ballooning (C), lobular inflammation (D), MASLD activity score (E), F4/80 positive cells (F), and fibrosis (G). mRNA expression of genes associated with fibrogenesis: *Col4a1* (H) and *Col4a2* (I). mRNA expression of genes associated with inflammation: *Il2* (J), *Il6* (K), *Ccl2* (L), *Ccl5* (M), *Cd68* (N), *Tnf* (O), and *Itgam* (P). Data are presented as mean ± SEM (n ≥ 6). ∗*P* < 0.05 versus chow, #*P* < 0.05 versus WD. Ccl, C-C motif chemokine ligand; Cd38, ADP-ribosyl cyclase; Col, collagen; Il, interleukin; Itgam, integrin subunit α-M; MASLD, metabolic dysfunction-associated steatotic liver disease; Tnf, tumor necrosis factor; WD, Western diet.

Histological scoring revealed that WD-fed WT mice had significantly increased liver steatosis, hepatocellular ballooning, and MASLD activity score compared to chow-fed animals (Fig. 2A–C, E). Similarly, all WD-fed KO mice exhibited the maximum score for liver steatosis, hepatocellular ballooning, lobular inflammation, and activity score (Fig. 2A–E). Hepatocellular ballooning and lobular inflammation were completely normalized by CDN1163 treatment in WT animals, and parallel effects were observed in KO animals (Fig. 2C, D). As a result, MASLD activity score was significantly reduced by CDN1163 treatment in both WT and KO WD-fed mice (Fig. 2E) despite insignificant reductions in liver steatosis (Fig. 2A, B). Furthermore, elevations in macrophage infiltration (F4/80 staining) due to WD feeding were significantly reduced by CDN1163 treatment in KO but not WT mice (Fig. 2F). Liver fibrosis was also significantly increased by WD feeding (Fig. 2G), which correlated with expression of collagen 4 genes (Fig. 2H, I). Importantly, CDN1163 treatment prevented the elevated expression of these fibrotic markers and maintained them at the basal level observed in chow-fed mice (Fig. 2G–I). Taken together, these data indicate that SERCA activation substantially improves liver tissue histology and inhibits fibrosis progression in obese, WD-fed animals.

### SERCA activation reverses WD-induced gene expression signatures associated with liver inflammation, oxidative stress, and ER stress

**Fig. 3:**
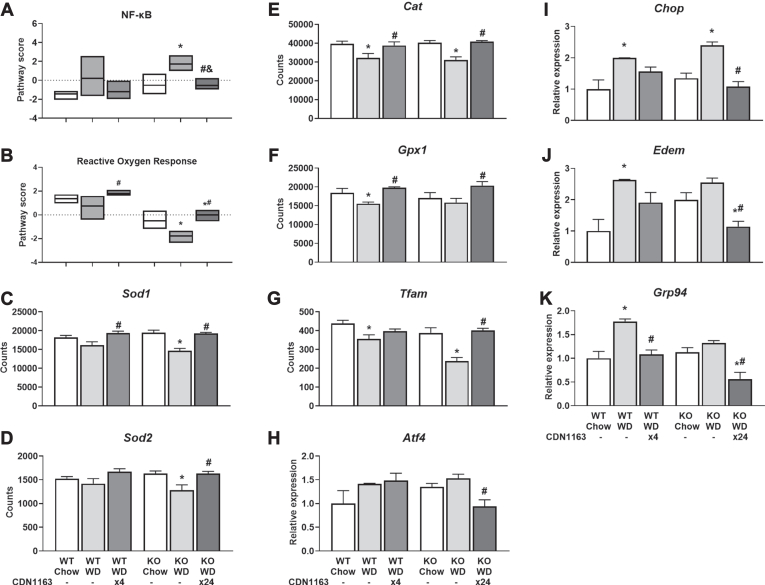
Activation of stress response pathways. NanoString pathway score of genes involved in NF-κB pathway signaling (A) and reactive oxygen response (B). mRNA expression (NanoString counts) of genes associated with antioxidant response: *Sod1* (C), *Sod2* (D), *Cat* (E), *Gpx1* (F), and *Tfam* (G). mRNA expression (real-time qPCR) of ER stress and UPR markers: *Atf4* (H) and *Chop* (I), *Edem* (J) and *Grp94* (K). Data are presented as mean ± SEM (n ≥ 6). ∗*P* < 0.05 versus chow, #*P* < 0.05 versus WD. NanoString mRNA data are reported as counts and real-time qPCR data are reported as relative expression. Atf4, activating transcription factor 4; Cat, catalase; Chop, C/EBP homologous protein; Edem, ER degradation enhancing α-mannosidase like protein 1; ER, endoplasmic reticulum; Gpx1, glutathione peroxidase 1; Grp94, heat shock protein 90 kDa β family member 1; qPCR, quantitative PCR; Sod, superoxide dismutase; Tfam, mitochondrial transcription factor A; UPR, unfolded protein response; WD, Western diet.

Next, we assessed whether CDN1163 treatment inhibited the upregulation of inflammatory, oxidative stress, and ER stress genes induced by WD feeding. CDN1163 decreased expression of interleukin 2 and 6 in liver tissue of KO mice (Fig. 2J, K). A variety of other inflammatory markers were upregulated by WD feeding and normalized by CDN1163 treatment in both WT and KO mice (Fig. 2L−P). In addition, CDN1163 decreased expression of genes involved in NF-κB signaling, which induces the expression of various proinflammatory genes, in WD-fed KO mice (Fig. 3A). Pathogenesis of MASLD has been linked to oxidative stress (25), and oxidative modification of SERCA modulates ER Ca^2+^ uptake and dysregulates intracellular Ca^2+^ homeostasis in hepatocytes, causing ER stress and subsequent MASLD and MASH (26). Therefore, we measured expression of genes encoding key antioxidant enzymes that defend against oxidative stress in the liver. WD feeding reduced reactive oxygen response (Fig. 3B) and antioxidant gene expression (Fig. 3C–F), an effect that was reversed by CDN1163 treatment. Oxidative damage can inhibit the transcription of critical mitochondrial proteins through repression of the transcription factor *Tfam*, which was downregulated by WD feeding and restored by CDN1163 treatment (Fig. 3G). Conversely, expression of genes involved in ER stress response and unfolded protein response were elevated by WD feeding and reduced by CDN1163 treatment (Fig. 3H−K). Additionally, CDN1163 normalized expression of genes involved in processes such as autophagy, DNA damage repair, endocytosis, lysosomal degradation, and nucleotide synthesis and salvage in WD-fed KO mice (supplemental Fig. S2), which might indicate decreased hepatocyte cell death and could explain the increasing trend in liver *Serca2* expression observed in CDN1163-treated animals (Fig. 1B). In summary, these results indicate that CDN1163 treatment mitigates the inflammatory responses of steatotic livers, which correlates to upregulation of antioxidant defense systems and reduction of ER stress.

### SERCA activation restores FA saturation in WD-fed mice by elevating liver PUFAs and reducing MUFAs

**Fig. 4:**
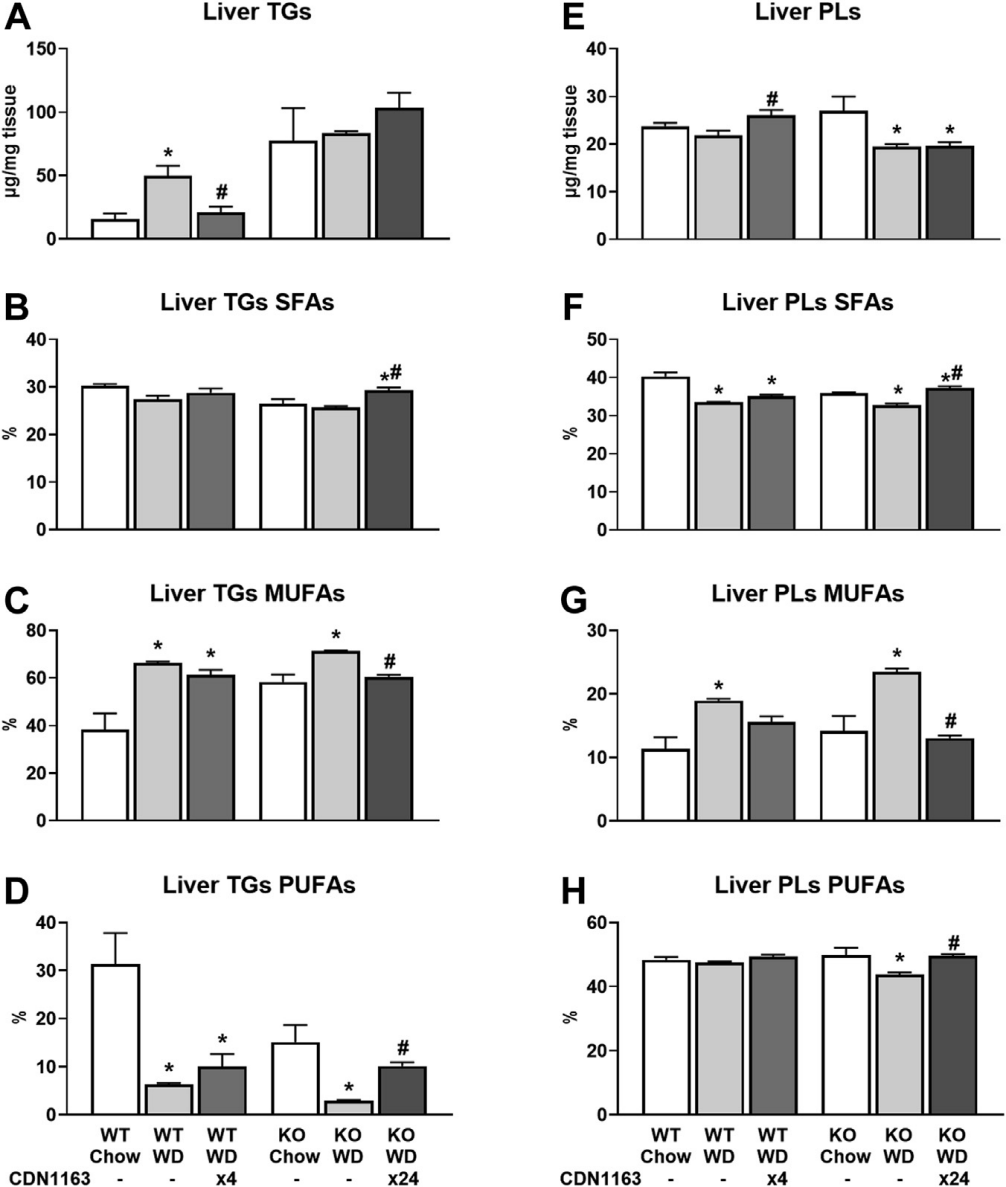
Absolute liver triglycerides and phospholipids, and their saturation. Hepatic triglycerides (A–D) and phospholipids (E–H) were measured using GC-FID, and their composition calculated as the percentage contribution of saturated, monounsaturated, and polyunsaturated fatty acids to each lipid species. Data are presented as mean ± SEM (n ≥ 6). ∗*P* < 0.05 versus chow, #*P* < 0.05 versus WD. FID, flame ionization detector; WD, Western diet.

**Fig. 5:**
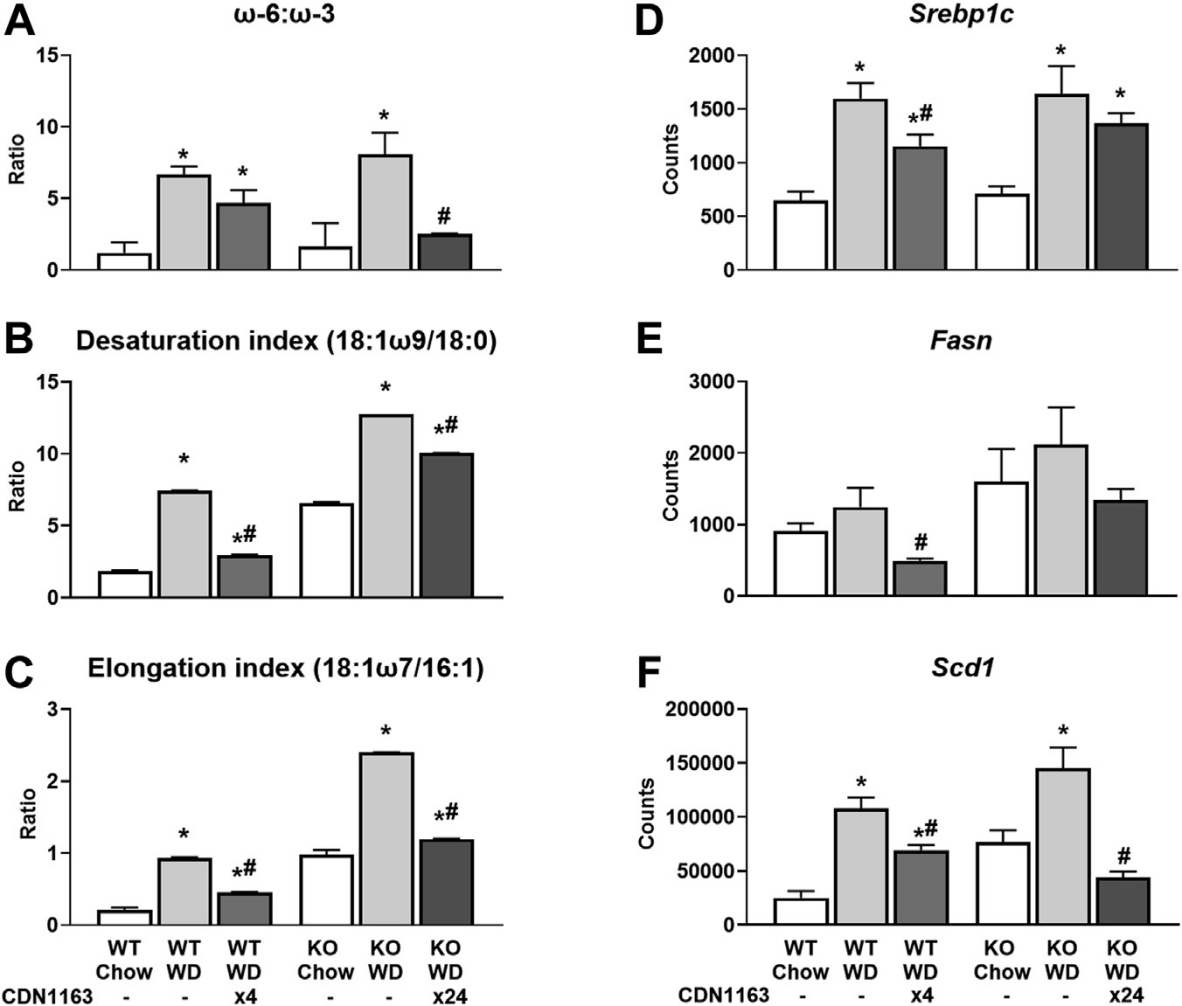
Fatty acid ratios and expression of lipid biosynthetic genes. Total lipids ω-6 to ω-3 PUFA ratio (A), desaturation index (B), and elongation index (C). mRNA expression of genes associated with fatty acid synthesis: *Srebp1c* (D), *Fasn* (E) and *Scd1* (F). Data are presented as mean ± SEM (n ≥ 6). ∗*P* < 0.05 versus chow, #*P* < 0.05 versus WD. Fasn, fatty acid synthase; Scd1, stearoyl-CoA desaturase 1; Srebp1c, sterol regulatory element binding protein 1c; WD, Western diet.

Since ER-localized enzymes synthesize the vast majority of cellular lipids, we investigated whether hepatic lipid composition was affected by CDN1163 treatment. Liver triglycerides (TGs) were increased by WD feeding and normalized to the basal, chow-fed levels by CDN1163 treatment in WT animals only (Fig. 4A). In contrast, TG content of KO livers was ∼5%–10% (w/w) under all conditions examined and was not affected by CDN1163 treatment. WD feeding altered the total abundance of liver phospholipids (PLs) and the saturation of FA side chains (Fig. 4E−H), which dramatically changes membrane biochemical properties and impairs SERCA function (8). The SFA content of PLs was decreased by WD feeding (Fig. 4F), while the MUFA content of TGs and PLs (Fig. 4C, G), as well as diacylglycerols and FFAs (supplemental Fig. S3C, G), was increased by WD. CDN1163 treatment opposed these WD-induced changes in FA composition. Expression of several genes associated with lipid biosynthesis (e.g., *Srebp1c*, fatty acid synthase (*Fasn*), and stearoyl-CoA desaturase 1 (*Scd1*)) and lipid transport (e.g., fatty acid translocase (*Cd36*) and apolipoproteins (*Apoa4*, *Apoc2*)) was upregulated by WD and lowered by CDN1163 (Fig. 5D–F, supplemental Figs. S4C, and S5A and B). Changes in SFA content were mainly caused by shifts in palmitic acid abundance (supplemental Tables S2–S6) that were also linked to altered expression of apolipoprotein *Apom* (supplemental Fig. S5C). Alterations in MUFA content were tightly connected with shifts in 18:1ω9 abundance (supplemental Tables S2–S6) that, in turn, were correlated with *Scd1* expression (Fig. 5F). Changes in Scd1 activity were further confirmed by calculating the desaturation index (18:1ω9 to 18:0 ratio) (Fig. 5B). Similarly, the expression of other desaturases (supplemental Fig. S4E, F) and proteins involved in FA elongation (supplemental Fig. S4G–J), as well as the elongation index (18:1ω7 to 16:1 ratio) (Fig. 5C), were increased by WD feeding and decreased by CDN1163.

The most pronounced shifts in liver FA composition, however, occurred with respect to PUFA species. A prior meta-analysis of randomized controlled trials provided substantial evidence that ω-3 PUFA supplementation has benefits for treating MASLD (27). Our work uncovered that PUFA content was decreased by WD feeding and increased by CDN1163 treatment across all lipid classes (Fig. 4D, H and supplemental Fig. S3D, H, L). The spike in PUFA content after CDN1163 treatment was mainly caused by increased linoleic acid (18:2) content (supplemental Tables S2–S6), but also increases in 20:4 content of PLs (supplemental Table S2), 20:5 content of ceramides (supplemental Table S6) and 22:6 content of both PLs and ceramides (supplemental Tables S2 and S6). It has been recently proposed that imbalances involving high ω-6 and low ω-3 PUFA intake from WDs contributes to MASLD pathogenesis in children with obesity (28). Indeed, WD feeding increased ω-6:ω-3 ratios, whereas CDN1163 treatment opposed this effect (Fig. 5A). Changes in PUFA levels positively correlated with expression of transmembrane fatty acid transporters responsible for trafficking long-chain PUFAs (supplemental Fig. S4K, L). These data suggest that CDN1163 shifts fatty acid composition from MUFAs to PUFAs by downregulating de novo lipogenesis while promoting transport and desaturation of ω-3 PUFAs.

### SERCA activation decreases liver CAC and pyruvate cycle fluxes

**Fig. 6:**
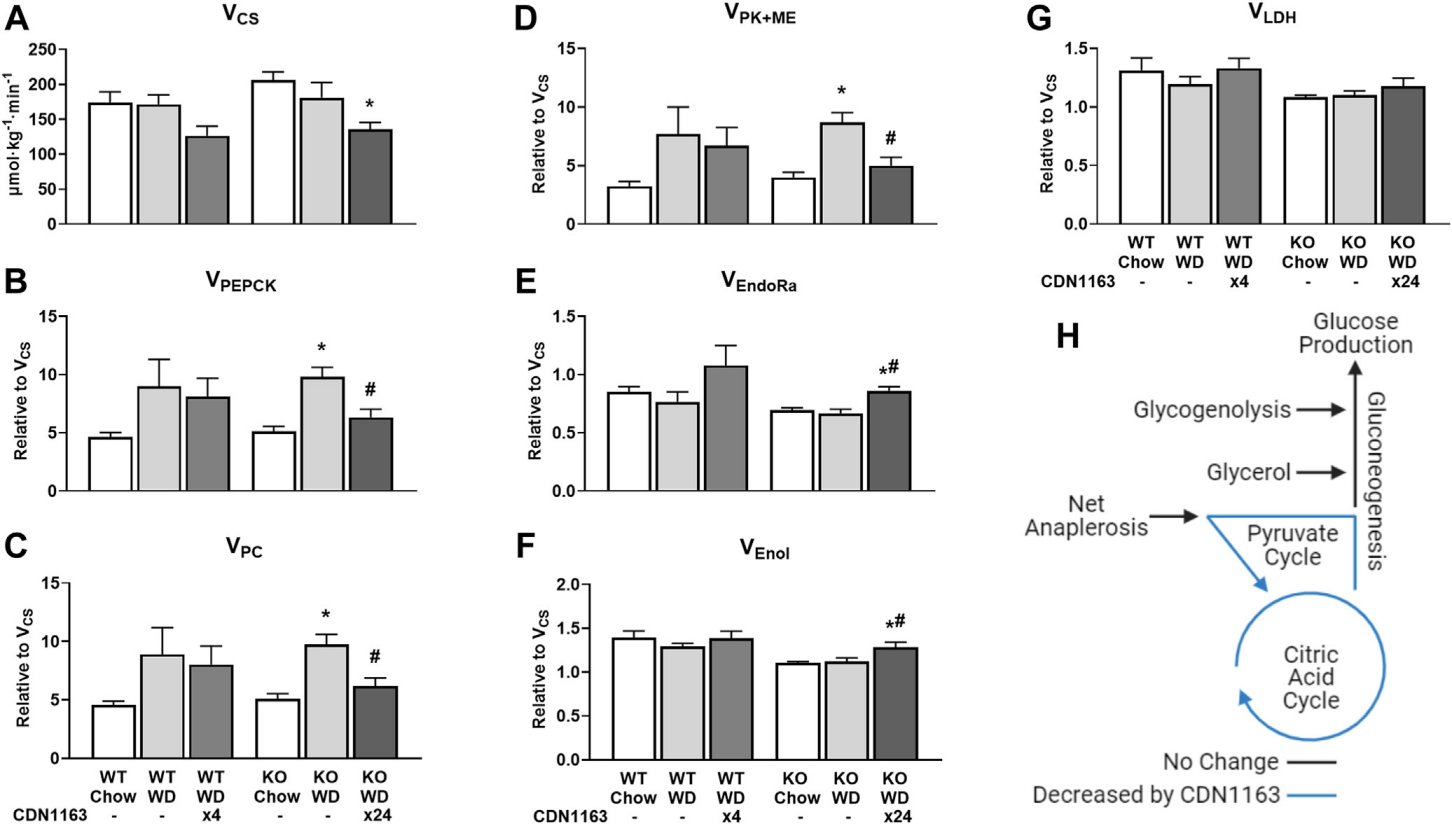
Effects of WD and CDN1163 treatment on in vivo metabolic fluxes. Metabolic flux analysis was used to estimate rates of enzyme catalyzed reactions in 20 h-fasted mice: absolute citrate synthase flux (A) and relative rates of pyruvate cycling (B–D), endogenous glucose production (E), gluconeogenesis from PEP (F), and net anaplerosis (G). Metabolic map depicting significant changes in absolute flux rates due to CDN1163 treatment of WD-fed KO mice (H). Rates of PEP carboxykinase (V_PEPCK_), pyruvate carboxylase (V_PC_), pyruvate kinase and malic enzyme (V_PK+ME_), enolase (V_Enol_), lactate dehydrogenase (V_LDH_), and endogenous glucose production (V_EndoRa_) are expressed relative to citrate synthase (V_CS_). Note that enolase flux is expressed in triose units. Fluxes were regressed using the enrichment of mass isotopomers of liver alanine, glutamate, lactate, and urea and plasma glucose, alanine, glutamine, and lactate obtained from the artery of conscious, unstressed mice during the isotopic steady state. Data are presented as mean ± SEM (n ≥ 6). ∗*P* < 0.05 versus chow, #*P* < 0.05 versus WD. CS, citrate synthase; EndoRa, endogenous glucose production; Enol, enolase; LDH, lactate dehydrogenase; ME, malic enzyme; PC, pyruvate carboxylase; PEP, phosphoenolpyruvate; PEPCK, phosphoenolpyruvate carboxykinase; PK, pyruvate kinase; WD, Western diet.

Changes in the composition of membrane PLs impairs SERCA activity in the obese liver (8) and alters transport of calcium ions between subcellular organelles (29). We have previously shown that dysregulation of intracellular calcium homeostasis promotes increased mitochondrial oxidative metabolism and accumulation of toxic ROS in palmitate-treated hepatocytes (4). Therefore, we performed in vivo ^13^C flux analysis to assess changes in hepatic metabolism in response to WD feeding and CDN1163 treatment. CDN1163 treatment decreased absolute rates of citrate synthase flux (V_CS_) in WD-fed animals (Fig. 6A), consistent with the hypothesis that limiting Ca^2+^ transport from ER to mitochondria prevents hyperactivation of mitochondrial metabolism in fat-laden hepatocytes (4). CDN1163 also restored basal expression of genes involved in gluconeogenesis, pentose phosphate pathway, FA synthesis and FA oxidation (supplemental Fig. S6A–D), as well as genes involved in mitochondrial respiration (supplemental Fig. S6E–L), the latter of which could contribute to lowering CAC-associated fluxes by improving substrate oxidation and redox energy coupling in liver mitochondria. WD-fed KO mice exhibited increased liver pyruvate cycling via V_PEPCK_, V_PC_, and V_PK+ME_ fluxes, similar to previous reports in obese mice (14) and humans with MASLD (30), which was normalized by CDN1163 treatment (Fig. 6B–D). Pyruvate cycling is an ATP-consuming cycle that becomes upregulated when the liver is in a high-energy state characteristic of overnutrition, possibly due to activation of pyruvate carboxylase by excess acetyl-CoA (31). Furthermore, CDN1163-treated KO mice had slightly elevated endogenous glucose production (V_EndoRa_) and enolase flux (V_Enol_) relative to V_CS_ (Fig. 6E, F), which indicates increased coupling between CAC and gluconeogenesis, while net anaplerosis (V_LDH_) was unaffected (Fig. 6G). However, V_EndoRa_ and V_Enol_ fluxes were unchanged by CDN1163 treatment when expressed as absolute rates. Overall, CDN1163 reduced mitochondrial CAC and pyruvate cycling fluxes (Fig. 6H), which have been previously implicated in promoting oxidative stress and inflammation in MASLD (32).

### SERCA activation shifts hepatocellular redox to a less oxidized state

**Fig. 7:**
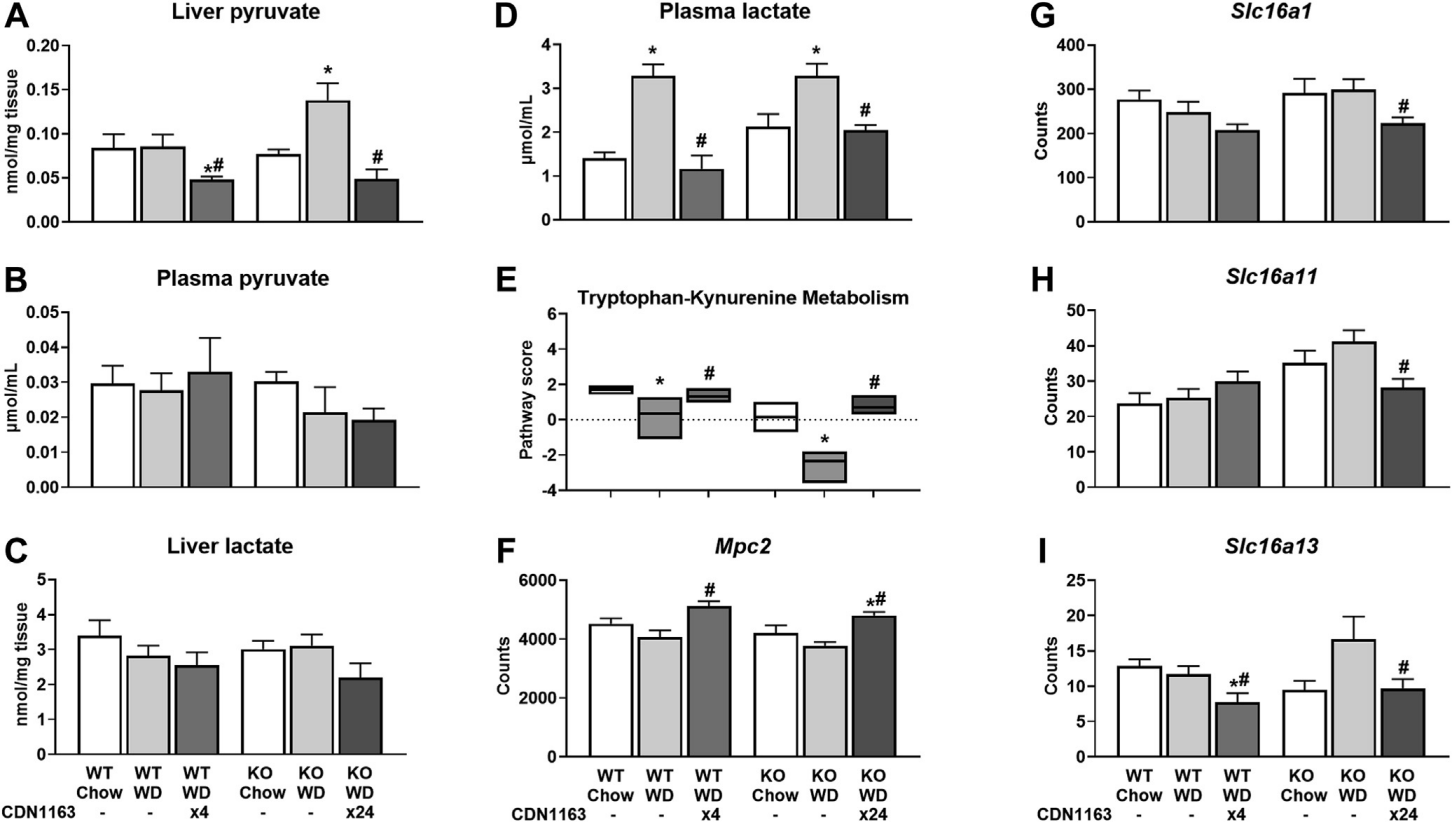
Pyruvate metabolism and transport. Pyruvate and lactate content of liver tissue and plasma samples (A–D). NanoString pathway score of tryptophan-kynurenine metabolism (E). mRNA expression of pyruvate transporter into mitochondria, *Mpc2* (F). (G–I) mRNA expression of transporters of monocarboxylates, such as lactate and pyruvate, across the plasma membrane: *Slc16a1* (G), *Slc16a11* (H), and *Slc16a13* (I). Data are presented as mean ± SEM (n ≥ 6). ∗*P* < 0.05 versus chow, #*P* < 0.05 versus WD. Mpc2, mitochondrial pyruvate carrier 2; Slc16, solute carrier family 16 member; WD, Western diet.

**Fig. 8:**
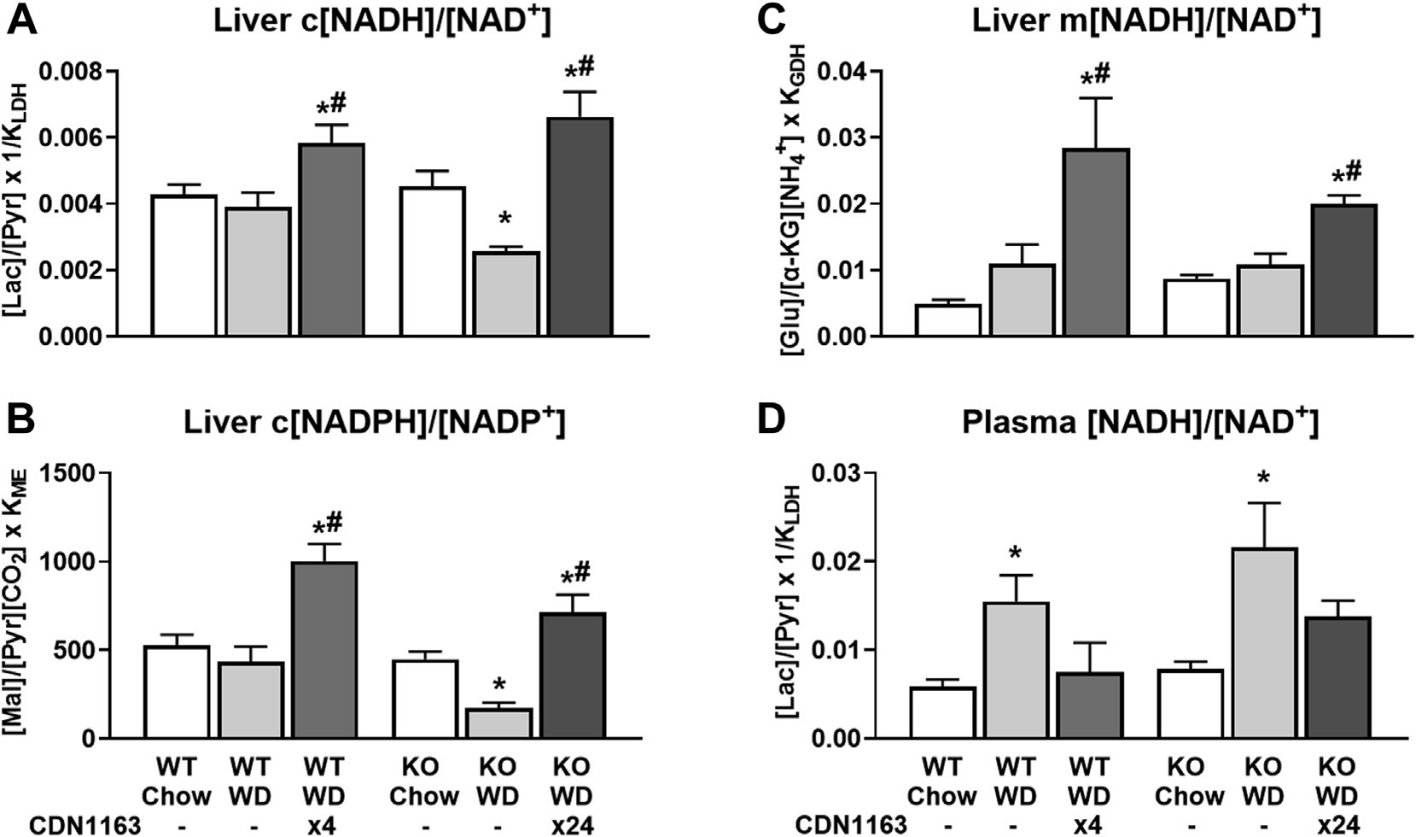
Cytosolic and mitochondrial redox state for liver (A–C) and plasma (D) estimated using enzymatic equilibrium relations: Liver c[NADH]/[NAD^+^] = [Lactate]/[Pyruvate] × K_LDH_, Liver c[NADPH]/[NADP^+^] = [Malate]/[Pyruvate][CO_2_] × K_ME_, Liver m[NADH]/[NAD^+^] = [Glutamate]/[α-ketoglutarate][NH_4_^+^] × K_GDH_, Plasma [NADH]/[NAD^+^] = [Lactate]/[Pyruvate] × K_LDH_. Data are presented as mean ± SEM (n ≥ 6). ∗*P* < 0.05 versus chow, #*P* < 0.05 versus WD. GDH, glutamate dehydrogenase; α-KG, α-ketoglutarate; LDH, lactate dehydrogenase; ME, malic enzyme; WD, Western diet.

To further investigate the causes and consequences of altered CAC and pyruvate cycling fluxes, we profiled liver metabolite content and transporter expression. Liver pyruvate was elevated in WD-fed KO mice, concomitant with higher pyruvate cycling, while CDN1163 treatment reduced pyruvate content in both WT and KO mouse livers (Fig. 7A). Plasma pyruvate did not vary significantly among the experimental groups (Fig. 7B). In contrast, liver lactate remained unchanged, whereas plasma lactate was increased by WD feeding and normalized by CDN1163 treatment (Fig. 7C, D). This observation pointed to changes in the redox state of liver and peripheral tissues. Indeed, liver cytosolic [NADH]/[NAD^+^] and [NADPH]/[NADP^+^] ratios as well as mitochondrial [NADH]/[NAD^+^] ratio shifted to a less oxidized state following CDN1163 treatment (Fig. 8A–C). Plasma [NADH]/[NAD^+^] ratio, which mainly reflects the redox status of peripheral tissues (33), exhibited an inverse relationship to liver c[NADH]/[NAD^+^]; the plasma ratio was significantly increased by WD feeding and normalized by CDN1163 action (Fig. 8D). Changes in liver redox state were correlated with expression of tryptophan-kynurenine pathway genes (Fig. 7E), responsible for de novo biosynthesis of NAD^+^, and expression of mitochondrial pyruvate carrier *Mpc2* (Fig. 7F) and plasma membrane monocarboxylate transporters (Fig. 7G–I). In summary, our data suggest that SERCA activation has pronounced effects on liver redox state and substrate supply, which may partially explain the observed changes in liver CAC and pyruvate cycling fluxes. These findings are especially relevant to MASLD treatment since gradual loss of mitochondrial function correlates with oxidative tissue damage in MASH patients (2). Changes in redox state and cellular ROS levels may also contribute to altered PUFA composition of liver lipids, since PUFA desaturation consumes NADH (34), and PUFA peroxidation provides a mechanism to scavenge free radicals (35).

## Discussion

Dysfunction of cellular organelles such as the ER and mitochondria has emerged as a key determinant of MASLD severity and a potential therapeutic target (36, 37, 38). In the livers of obese animals, ER membrane composition is altered and its capacity to retain Ca^2+^ is impaired (8). Ca^2+^ is an important second messenger that stimulates energetic metabolism in many tissues. Ca^2+^ released by the ER is readily taken up by the mitochondria due to their proximity to the ER membranes. Once in the mitochondria, Ca^2+^ ions potently activate several mitochondrial enzymes and transporters critical for oxidative metabolism (39). Our lab (4) and other groups (29) have shown that disrupting Ca^2+^ transport from the ER to mitochondria can restore the basal mitochondrial metabolic activity and reduce oxidative stress in fat-laden hepatocytes. Therefore, we hypothesized that enhancing SERCA activity in vivo would promote ER Ca^2+^ retention in hepatocytes and decelerate MASLD progression. We tested this hypothesis by treating MASLD mouse models with the small-molecule allosteric SERCA activator CDN1163 during an 8-week period of WD feeding. One prior study reported that short-term treatment of *ob/ob* mice with CDN1163 over five consecutive days improved liver steatosis, reduced hepatic expression of lipogenic and ER stress-induced genes, and increased expression of respiratory and oxidative defense genes (10). While our study confirmed similar hepatoprotective outcomes, we demonstrate that prolonged CDN1163 treatment can limit progression from MASLD to MASH and prevent hyperactivation of mitochondrial fluxes due to chronic WD feeding.

CDN1163 treatment of WD-fed mice restored whole-body indices of glucose tolerance and insulin sensitivity to those of chow-fed mice, which correlated with significant improvements in liver inflammation, fibrosis, and other histological markers of MASH. Among these, fibrosis is most directly related to clinical outcomes in MASLD patients (40). Despite limited effects on liver steatosis or TG content in KO mice, CDN1163 reduced hepatic CAC and pyruvate cycling fluxes, enhanced expression of mitochondrial respiratory genes, and shifted hepatocellular [NADH]/[NAD^+^] and [NADPH]/[NADP^+^] ratios to a less oxidized state. These results point to dramatic resolution of WD-induced MASH markers, metabolic dysfunction, and redox imbalance due to treatment with CDN1163. This was also associated with increases in PUFA content of liver lipids, which is an indicator of a reduced cytosolic redox state (34) and diminished ROS levels (35). We found that linoleic acid content was restored or even elevated by CDN1163 treatment, while the ω-6 to ω-3 PUFA ratio was reduced. Elevations in PUFA content have been linked to mitochondrial function recovery, reduction in ROS levels, mitigation of ER stress (41), and improvements in biochemical, inflammatory, and histopathological parameters of MASH in animals (42, 43, 44) and humans (27). Even though food intake was not impacted by CDN1163 treatment in either WT or KO mice, we observed modest, yet significant, reductions in fat mass and body weight that were dose-dependent in the case of KO mice. These findings could reflect an increase in whole-body energy expenditure caused by simultaneous activation of SERCA in various tissues, especially skeletal muscle (45).

Previously, stable isotopes were applied to determine in vivo metabolic fluxes in human subjects with either high or low intrahepatic TG content, and mitochondrial oxidative metabolism was found to be approximately 2-fold greater in the livers of MASLD patients (30). This increase in mitochondrial activity was associated with a 30% higher rate of gluconeogenesis and 50% higher rates of hepatic anaplerosis and pyruvate cycling, demonstrating that hepatic steatosis increases both energy-producing and energy-consuming fluxes in livers of MASLD patients. High-fat fed mice exhibit similar metabolic alterations, which correlate to oxidative stress, inflammation, and tissue injury (14, 46). Liver respiratory capacity is significantly reduced in MASH patients, despite having higher mitochondrial mass, due to accumulation of oxidative damage (2). Therefore, uncoupling of mitochondrial metabolism is hypothesized to be a key factor controlling progression from MASLD to MASH. However, metabolic energy can also be dissipated through mechanisms that do not involve mitochondrial uncoupling. Such mechanisms include so-called futile cycles that consume ATP without net conversion of substrate to product, thus releasing energy as heat. The SERCA-mediated Ca^2+^ import/export cycle is one such example, and activation of SERCA has been proposed as a strategy to counteract obesity by dissipating excess energy (47). Because CDN1163 increases energy metabolism in muscle cells (45), it is possible that CDN1163 treatment partially relieves metabolic burden on the liver by enhancing substrate oxidation in the periphery.

While pyruvate cycling has also been considered a type of futile cycle, recent studies suggest that it has a role in balancing liver metabolism across different subcellular compartments. Satapati *et al.* (32) showed that limiting pyruvate cycle flux through knockdown of liver phosphoenolpyruvate carboxykinase (PEPCK) promoted a more reduced cytosolic and mitochondrial redox state and protected mice from hepatic oxidative stress and inflammation during high-fat feeding. Knocking out pyruvate carboxylase, another enzyme involved in liver pyruvate cycling, had opposite effects on hepatic redox state and exacerbated diet-induced oxidative stress and inflammation (48). Our data indicate that CDN1163 phenocopies the effects of PEPCK knockdown on liver redox state and pyruvate cycling, while similarly limiting diet-induced inflammation and hepatocellular damage. CDN1163 treatment also altered pyruvate delivery to the liver as indicated by decreased abundance of liver pyruvate and plasma lactate, increased *Mpc2* expression, and decreased monocarboxylate transporter expression, the latter of which is metabolically protective in mice (49, 50). Therefore, our study contributes to the growing evidence that normalization of pyruvate cycling flux and redox balance in the liver is a potential focal point to improve metabolic phenotypes associated with MASLD pathogenesis.

Interestingly, changes in liver metabolic fluxes and redox state were decoupled from changes in hepatic TG content in KO mice. Similar results were reported in the previous study of Hasenour *et al.* (14), where 8 weeks of WD feeding elevated pyruvate cycling, but 20 weeks of WD extensively upregulated liver gluconeogenic flux and oxidative CAC metabolism, and worsened liver fibrosis, without further increases in liver TG content. Taken together, these results indicate that MASLD involves progressive changes at the molecular level that do not necessarily correlate with total liver lipid content. Previous studies have indicated that dietary fat and circulating fatty acids provide the greatest contributions to hepatic steatosis under conditions of obesity and insulin resistance (51), which could explain why liver TG content in KO mice was unresponsive to changes in lipogenic gene expression. In contrast, metabolic flux changes due to CDN1163 treatment were correlated to changes in liver lipid composition (e.g., increased PUFA content) as opposed to total lipid content. We hypothesize that the observed increase in [NADPH]/[NADP^+^] ratio and expression of antioxidant enzymes, along with changes in specific FA transporters, were responsible for the effects of CDN1163 to restore PUFA content of liver lipids. PUFAs have been previously shown to contribute hepatoprotective effects by suppressing de novo lipogenesis (52, 53), activating fatty acid oxidation (54), and inhibiting inflammation and fibrosis in the liver (55).

There are some limitations to the study design that impact our conclusions. First, CDN1163 activates SERCA in a variety of cell types both inside and outside the liver, with no evidence of isoform specificity. The molecule was first identified through extensive fluorescence resonance energy transfer screening of compounds that have the ability to reverse SERCA2a inhibition by its cardiac regulator, phospholamban (11). Since then, it has been proposed as a potential treatment for several diseases involving dysregulation of intracellular Ca^2+^ transport in the brain, peripheral nerves, lung, heart, skeletal muscle, and liver. A key goal of future studies will be to elucidate the molecular mechanisms and cellular targets that are responsible for its hepatoprotective effects. Second, melanocortin-4 receptor (*Mc4r*) KO may have confounding effects on liver metabolism. We chose to use *Mc4r*^*−/−*^ mice as a genetic model because they rapidly develop human-like MASH when placed on WD. In contrast to other genetic mouse models such as *ob/ob* (leptin-deficient) or *db/db* (deficient leptin signaling), loss of Mc4r is expected to have only indirect effects on liver metabolism (56). Interestingly, *MC4R* mutations are the most frequent monogenic cause of obesity in humans (13, 57), and common *MC4R* variants are linked to polygenic obesity in the general population (58). Finally, we chose to expose *Mc4r*^*−/−*^ and WT animals to an 8-week experimental dietary period. While 8 weeks of WD was sufficient to induce histological features of MASH and elevate pyruvate cycling flux in KO mice, it was insufficient to extensively upregulate liver glucose and oxidative metabolism as demonstrated in our prior study with 20 weeks of WD feeding (14).

Overall, this study found that pharmacological SERCA activation with CDN1163 dramatically reduced the lipotoxic effects of WD feeding and restored metabolic function in livers of obese mice. CDN1163 treatment improved diet-induced steatohepatitis by restoring glucose tolerance and insulin sensitivity; reducing liver fibrosis, inflammation, oxidative stress and ER stress; normalizing hepatic pyruvate cycling; enhancing expression of mitochondrial respiratory genes; and elevating PUFA content in hepatocytes, which correlated to improvement of histological markers of MASH. Prior research has convincingly shown that ER stress (59) and mitochondrial dysfunction (32) contribute to MASLD progression. Therefore, the observed improvements in ER/mitochondrial function are likely to be involved in mediating the hepatoprotective effects of CDN1163 treatment. However, the exact molecular mechanisms explaining the connections between SERCA activation by CDN1163 and changes in mitochondrial function, reductions in ER stress, and protection from inflammation and fibrosis in the liver are still incompletely understood. We further acknowledge that changes in peripheral adiposity and whole-body energy state could contribute to the effects of CDN1163 on liver phenotypes. Since intracellular calcium is a key regulator of many important physiologic functions, including muscle contraction, hormone secretion, glycogen metabolism, and cell division, it is difficult to pinpoint specific pathways responsible for the hepatoprotective effects of CDN1163 at this stage of investigation. Teasing apart the contributions from direct versus indirect effects of SERCA activation on liver phenotypes requires further tissue-specific gain/loss of function studies that will be a major topic of our future studies.

## Data availability

This study includes no data deposited in external repositories. All data are contained within the article and the accompanying Supplementary Material.

## Supplemental data

This article contains supplemental data (60, 61, 62, 63, 64, 65, 66).

## Conflict of interest

The authors declare that they have no conflicts of interest with the contents of this article.

## Supplemental data

Supplemental Figures, tables, methods, references
