# Supplementary material for: Pharmacological SERCA activation limits diet-induced steatohepatitis and restores liver metabolic function in mice
Source: J Lipid Res. 2024 May 8;65(6):100558. doi: 10.1016/j.jlr.2024.100558 (PMC11179628; doi:10.1016/j.jlr.2024.100558)
Supplement: Supplemental Figures, tables, methods, references [file mmc1.docx]

**SUPPLEMENTARY MATERIAL to accompany the research article:**

**Pharmacological SERCA activation limits diet-induced steatohepatitis and restores liver metabolic function in mice**

Tomasz K Bednarski^1^, Mohsin Rahim^1^, Clinton M Hasenour^1^, Deveena R Banerjee^2^, Irina A Trenary^1^, David H Wasserman^2^, Jamey D Young^1,2^

^1^Department of Chemical and Biomolecular Engineering and ^2^Department of Molecular Physiology and Biophysics, Vanderbilt University, Nashville, Tennessee, USA.

**SUPPLEMENTAL methods**

**Glucose tolerance tests**

Animals were fasted for 6 hours starting at the beginning of the light cycle. At 12 pm, fasting plasma glucose concentrations were determined using an Accu-Chek glucometer (Roche, Basel, Switzerland). Immediately after, mice were given an IP injection of glucose (2 g/kg). Glucose levels were measured from tail incision at 15, 30, 60, 90 and 120 minutes.

***In vivo* isotope tracer infusions**

Mice were fasted 16 hours overnight prior to tracer infusions. In the morning, mice received an intravenous primed (1.5 µCi) continuous (0.075µCi/min) infusion of [3-^3^H]glucose. An hour later, mice were given a primed (200 µmol/kg) continuous (50 µmol/kg/min) infusion of sodium [^13^C_3_]lactate (Cambridge Isotope Laboratories, Tewksbury, MA), which persisted for another 2 hours to allow isotopic labeling to equilibrate. Arterial plasma glucose samples were obtained just before the start of the [^13^C_3_]lactate infusion (to provide a baseline measurement) and right before the end of the infusion. At the end of this 3-hour infusion, mice were immediately euthanized through cervical dislocation and tissues were rapidly excised and freeze-clamped in liquid nitrogen; plasma samples and tissues were stored at -80°C prior to analysis.‬‬‬‬‬‬‬‬‬‬‬‬‬‬‬

‬‬‬‬‬‬‬‬‬‬‬‬‬‬‬‬‬‬‬‬‬‬‬‬‬‬‬‬‬‬‬‬‬‬‬‬‬‬‬‬‬‬‬‬‬‬‬‬‬‬‬‬‬‬‬‬‬‬‬‬‬‬‬‬‬‬‬‬‬‬‬‬‬‬‬‬‬‬‬‬‬‬‬‬‬ **Metabolite extraction and GC-MS analysis**

Plasma samples were subjected to a chemical conversion of glucose to its di-*O*-isopropylidene propionate (DiO-Pr), aldonitrile pentapropionate (Aldo-Pr) or methyloxime pentapropionate (Mox-Pr) derivative. Additionally, some plasma samples underwent a biphasic methanol/water/chloroform extraction and a two-step chemical conversion of metabolites to their methyloxime *tert*-butyldimethylsilyl (Mox-TBDMS) derivatives using MBTSTFA + 1% TBDMCS (ThermoFisher Scientific, Waltham, MA). Liver samples were initially homogenized in methanol/water/chloroform using a bead homogenizer, and the extracted metabolites were subsequently converted to Mox-TBDMS derivatives. Glucose and other metabolite derivatives were analyzed with an Agilent 7890A gas chromatograph equipped with an HP-5ms capillary column and 5975C mass spectrometer in scan mode for analysis of metabolite pool size and isotopic enrichment.

**Liver metabolic flux analysis (MFA)**

Mass isotopomer distributions (MIDs) for plasma metabolites (glucose, alanine, lactate, and glutamine) and liver metabolites (alanine, lactate, glutamate and urea) were modeled with INCA to obtain best-fit flux estimates. Relative fluxes were determined for each mouse by constraining citrate synthase flux (V_CS_) to an arbitrary value of 100 and then minimizing the sum of squared residuals (SSRs) between model-simulated and experimentally measured MIDs. Measurement errors were specified to be either 0.4 mol% or the SEM of technical replicates, whichever was greater. Best-fit flux estimates were obtained through least-squares regression using a minimum of 50 random initial parameter sets. Goodness-of-fit was assessed using a chi-square test, and 95% flux confidence intervals were calculated by evaluating the sensitivity of the SSR to variations in flux values (1). Endogenous glucose production (EndoR_a_) and glucose disposal rates were determined using Steele’s non–steady-state equations, as described previously (2). The rate of EndoR_a_ was calculated in molar units (μmol∙kg^-1^∙min^-1^) which was used to convert relative hepatic fluxes for each mouse to absolute flux rates (i.e., in units of μmol∙kg^-1^∙min^-1^).

**Liver histology**

H&E-stained liver tissues were scored for the extent of steatosis (0-3), lobular inflammation (0-3) and hepatocellular ballooning (0-2), with the aggregate of the three scores equating to the MASLD activity score. Fibrosis was assessed with picrosirius red staining and assigned a separate score (0-4). Macrophage penetration was determined by immunohistochemical staining with F4/80 antibody (NB600-404; Novus Biologicals LLC, Littleton, CO). Briefly, slides were visualized with the Pannoramic 250 Flash III digital scanner (3DHISTECH Ltd., Budapest, Hungary). Quantification of immunolabeled cells in livers was performed in QuPath version 0.2.3, an open source digital pathology platform (3), using simple tissue detection to identify the region of interest. Positive pixels were detected using the Train Pixel Classifier function, employing an artificial neural network classifier at 1.56 µm/px resolution. Macrophages and Kupffer cells that demonstrated positive membranous and cytoplasmic labelling with F4/80 staining were manually selected to aid in training of positive pixels, while unlabeled hepatocytes were manually selected to aid in training of negative pixels. Data were collected as percent positive pixels per unit area.

**Liver lipid profiling**

Liver lipids were separated using Silica Gel 60A plates developed in petroleum ether, diethyl ether, and acetic acid (80:20:1). Plates for ceramide analysis were re-chromatographed in heptane, isopropyl ether and acetic acid (60:40:3). Lipids were visualized with rhodamine 6G; free fatty acids (FFAs), diacylglycerides (DAGs), triglycerides (TGs), phospholipids (PLs) and ceramides were scraped from plates, transmethylated with BF_3_/methanol and analyzed with an Agilent 7890 gas chromatograph equipped with an SP2380 column and flame ionization detector (FID). The retention times of known standards were used to identify fatty acid methyl esters; quantification was achieved through a comparison to margaric acid (17:0) that was included as internal standard in the analysis.

**Quantification of tissue and plasma metabolites**

Approximately 30 mg of liver tissue or 30 μL of plasma was spiked with 20 μL of 5 mM norvaline (internal standard) followed by a biphasic methanol/water/chloroform extraction (4). Similarly, calibration curve standards with known amounts of pyruvate, lactate and internal standard were extracted simultaneously for absolute quantification. The aqueous phase was dried, derivatized with Mox-TBDMS, and metabolites were quantified using a GC-MS instrument, as described above. For analysis of ammonium, 300-µL samples were diluted with dH_2_O to 1.5 mL and then analyzed using an Ammonia Gas Sensing Electrode (Thermo Fisher Scientific, MA) according to the user manual.

**Redox calculations**

Cytosolic NADH/NAD^+^ was estimated from lactate dehydrogenase equilibrium (cNADH/NAD^+^ = [Lactate]/[Pyruvate] x K_LDH_; where K_LDH_ = 1.11 x 10^-4^). Similarly, cytosolic NADPH/NADP^+^ was estimated from malic enzyme equilibrium (cNADPH/NADP^+^ = [Malate]/[Pyruvate][CO_2_] x K_ME_; where K_ME_ = 3.44 x 10^-2^ M and the CO_2_ concentration was taken to be 1.16 mM). Lastly, mitochondrial NADH/NAD^+^ was estimated from glutamate dehydrogenase equilibrium (mNADH/NAD^+^ = [Glutamate]/[α-ketoglutarate][NH_4_^+^] x K_GDH_; where K_GDH_ = 3.87 x 10^-6^ M) (5).

**Analysis of mRNA expression by quantitative real-time PCR**

In order to perform quantitative real-time PCR analysis, cDNA was synthesized using the iScript cDNA synthesis kit (Bio-Rad, Hercules, CA). cDNA was then diluted 1:25 with DI water, mixed with custom-made 5 µmol sets of primers (Integrated DNA Technologies, Coralville, IA) and iQ SYBR Green Supermix (Bio-Rad, Hercules, CA), then analyzed on a CFX96 Real-Time PCR System (Bio-Rad, Hercules, CA). Relative gene expression was normalized to peptidylprolyl isomerase A (*Ppia*) (6) using the 2^-ΔΔCt^ method.

**Analysis of mRNA expression by NanoString nCounter system**

Gene expression and pathway enrichment analysis was performed using the NanoString murine nCounter Metabolic Pathways Panel (NanoString Technologies, Seattle, WA) according to the manufacturer’s instructions. Briefly, raw gene expression data was analyzed using NanoString's software nSolver V.4.0 with the Advanced Analysis 2.0 plugin. Data normalization was performed using internal negative and positive controls and 20 selected housekeeping genes that were identified using the geNorm algorithm (<https://genorm.cmgg.be/>). Genes were clustered into pathways according to classifications provided by the Kyoto Encyclopedia of Genes and Genomes (KEGG). Pathway scores were calculated as the first principal component of the pathway’s normalized gene expression (7). The NanoString platform was also used to count individual mRNA transcripts.

**SUPPLEMENTAL FIGURES**


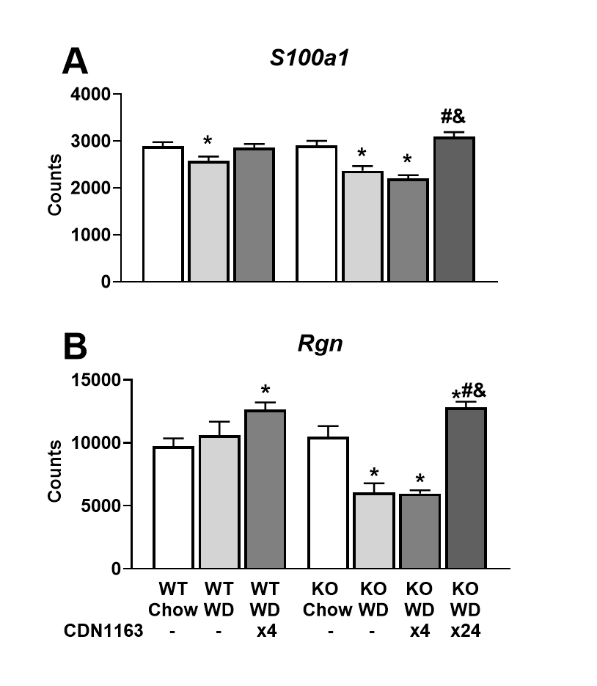


Fig. S1. mRNA expression of genes involved in regulation of calcium homeostasis: *S100a1* (A) and *Rgn* (B). Data are presented as mean ± SEM (n ≥ 6). **p* < 0.05 versus chow, #*p* < 0.05 versus WD and &*p* < 0.05 versus WD CDN1163 x4.


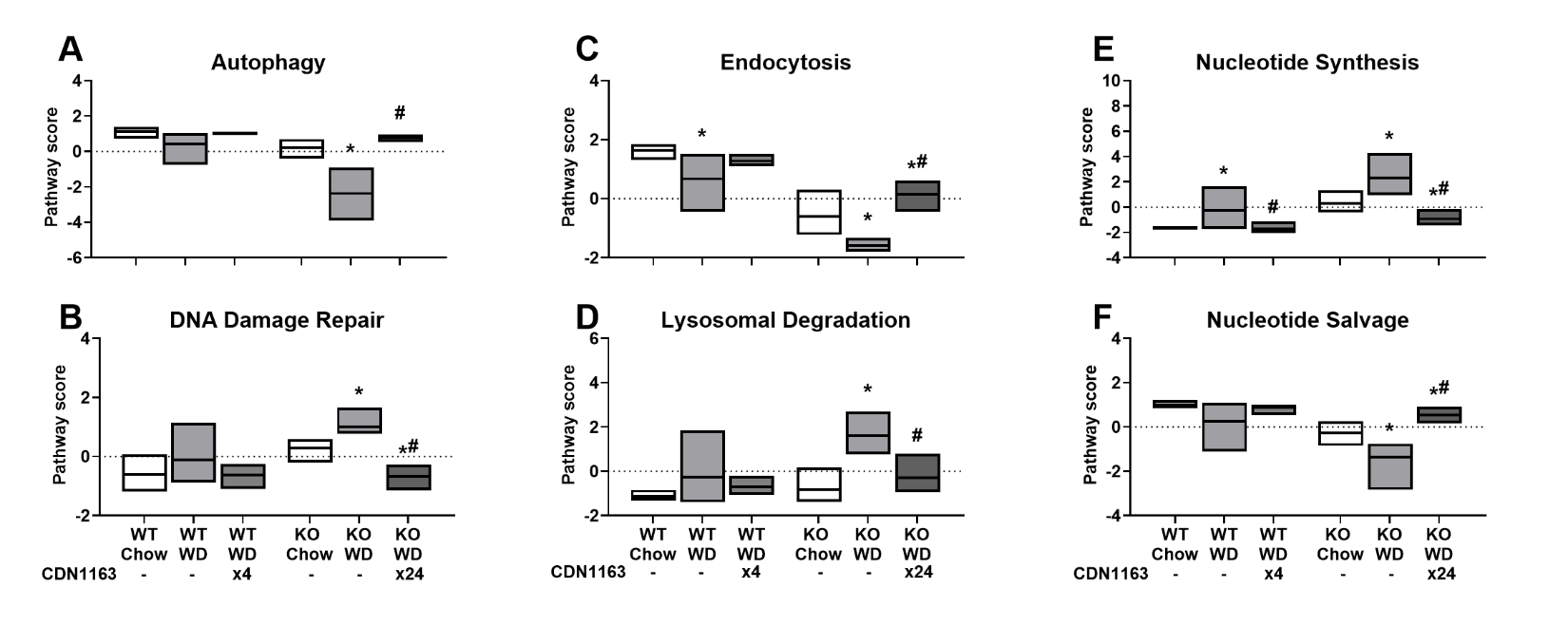


Fig. S2. NanoString pathway score of genes involved with autophagy (A), DNA damage repair (B), endocytosis (C), lysosomal degradation (D), nucleotide synthesis (E), and nucleotide salvage (F). Data are presented as mean ± SEM (n ≥ 6). **p* < 0.05 versus chow, #*p* < 0.05 versus WD.


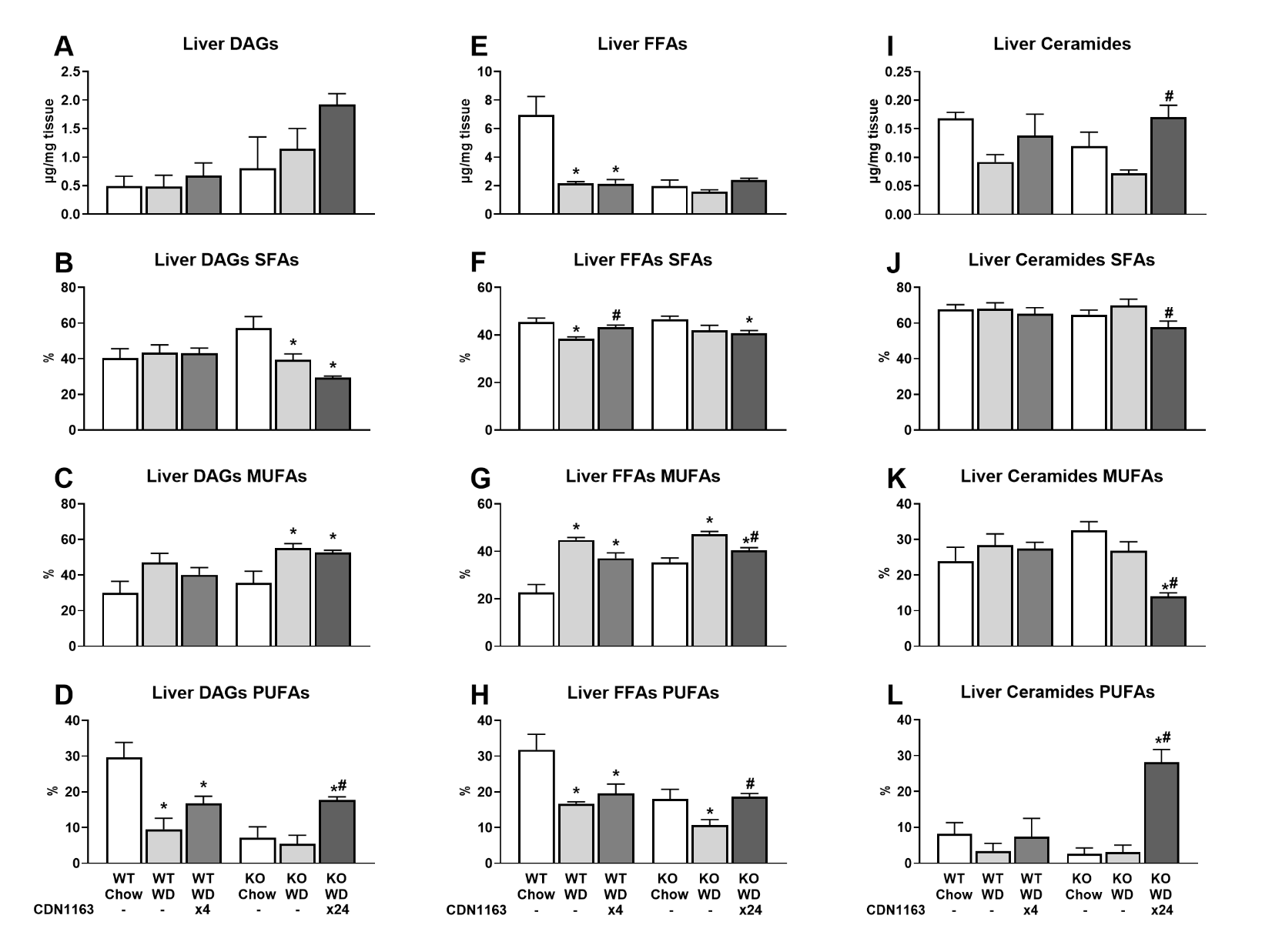


Fig. S3. Absolute liver diacylglycerides (A-D), free fatty acids (E-H), and ceramides (I-L), and their saturation. Data are presented as mean ± SEM (n ≥ 6). **p* < 0.05 versus chow, #*p* < 0.05 versus WD.


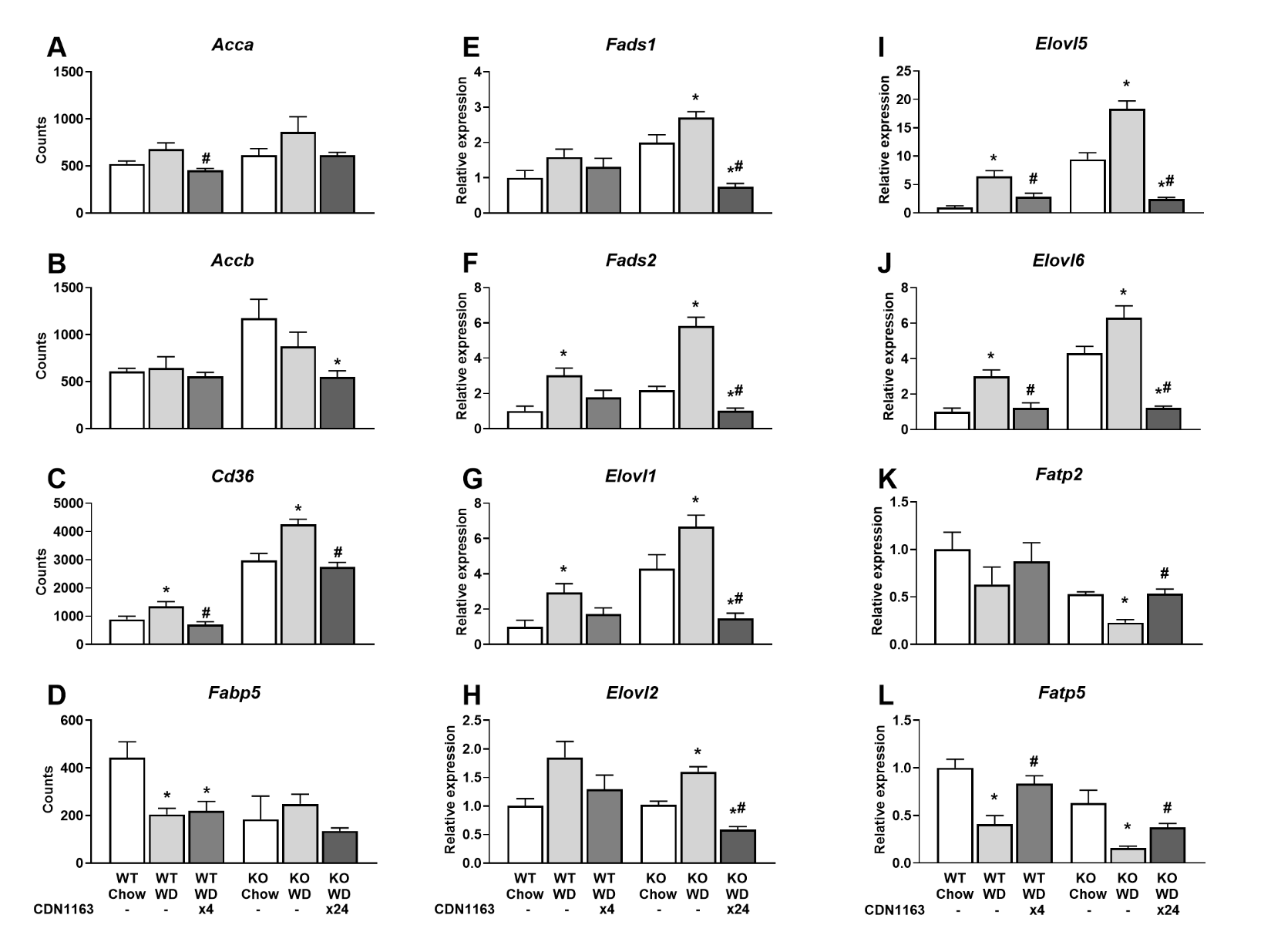


Fig. S4. mRNA expression of genes associated with fatty acid metabolism and transport: *Acca* (A), *Accb* (B), *Cd36* (C), *Fabp5* (D), *Fads1* (E), *Fads2* (F), *Elovl1* (G), *Elovl2* (H), *Elovl5* (I), *Elovl6* (J), *Fatp2* (K) and *Fatp5* (L). Data are presented as mean ± SEM (n ≥ 6). **p* < 0.05 versus chow, #*p* < 0.05 versus WD. NanoString mRNA data are reported as counts and real-time qPCR data are reported as relative expression.


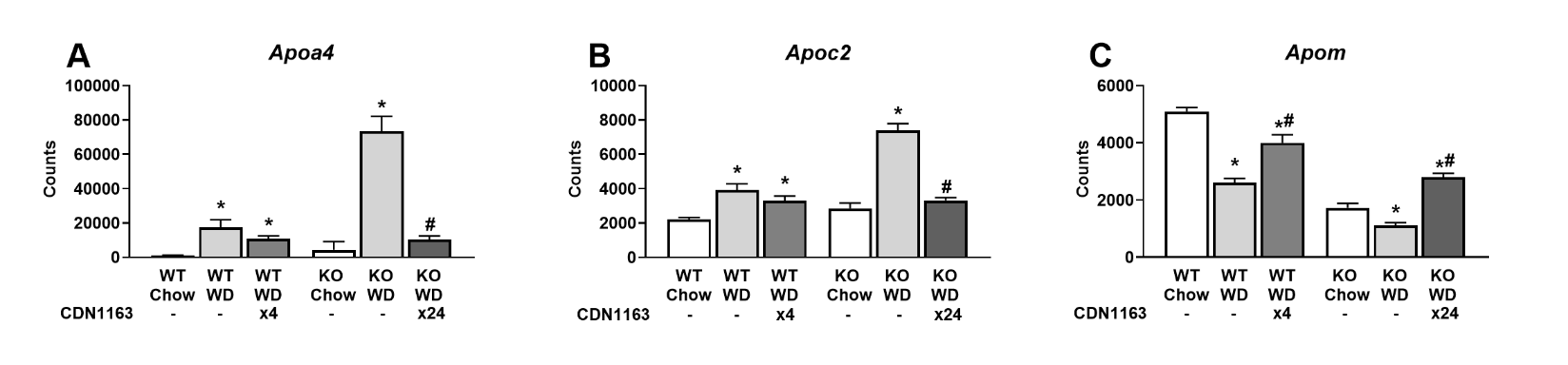


Fig. S5. mRNA expression of apolipoproteins: *Apoa4* (A), *Apoc2* (B) and *Apom* (C). Data are presented as mean ± SEM (n ≥ 6). **p* < 0.05 versus chow, #*p* < 0.05 versus WD.


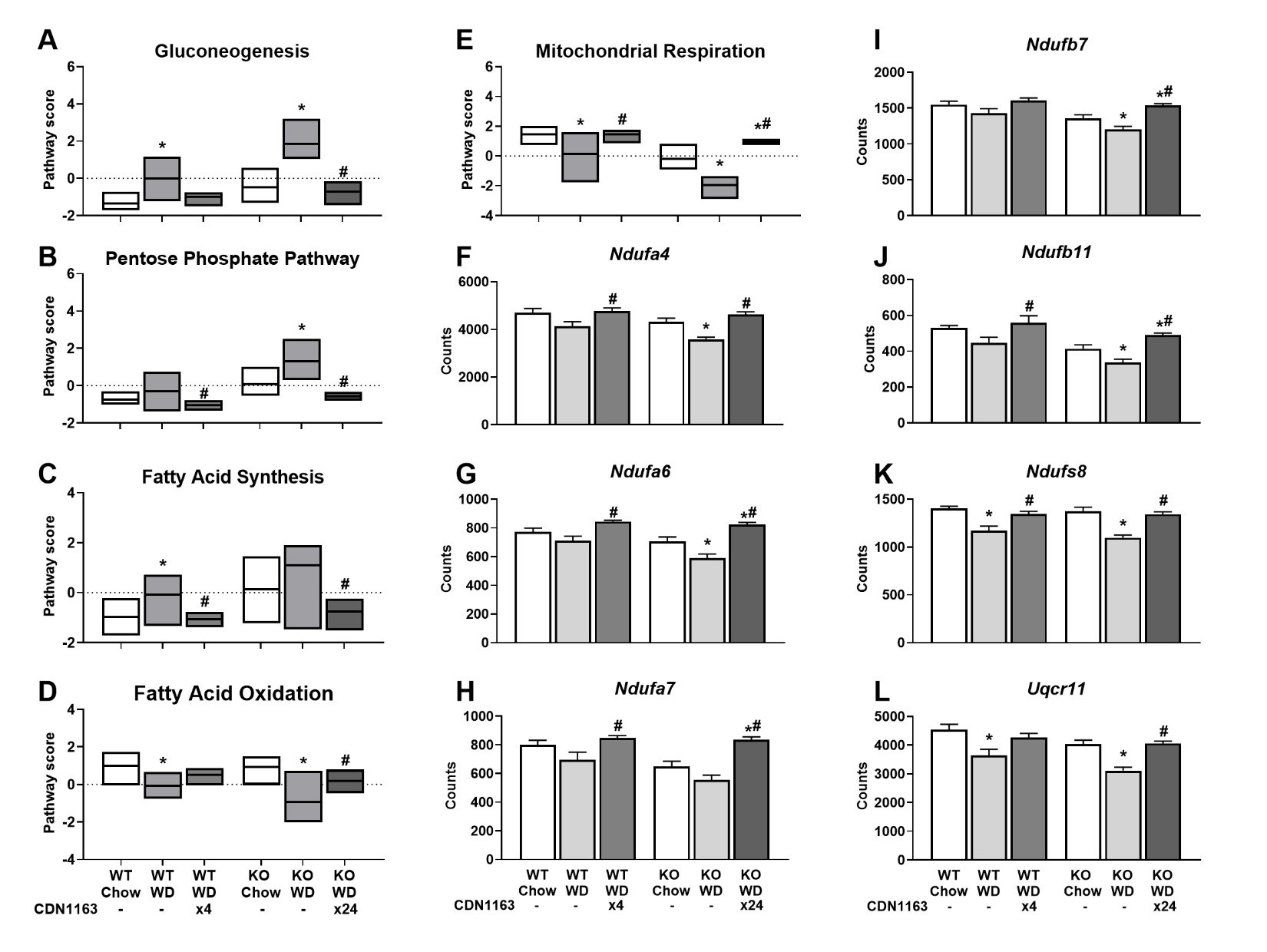


Fig. S6. NanoString pathway score of genes involved with gluconeogenesis (A), pentose phosphate pathway (B), fatty acid synthesis (C), fatty acid oxidation (D), and mitochondrial respiration (E). mRNA expression of genes associated with mitochondrial respiratory chain: *Ndufa4* (F), *Ndufa6* (G), *Ndufa7* (H), *Ndufb7* (I), *Ndufb11* (J), *Ndufs8* (K) and *Uqcr11* (L). Data are presented as mean ± SEM (n ≥ 6). **p* < 0.05 versus chow, #*p* < 0.05 versus WD.

**SUPPLEMENTAL TABLES**

| **Flux** | **Reaction Network** |
| --- | --- |
| **Hepatic compartment** |  |
| **V_EndoRa_** | G6P.L (ABCDEF) → Glucose (ABCDEF) |
| **V_Aldo.L_** | DHAP.L (CBA) + GAP.L (DEF) → G6P.L (ABCDEF) |
| **V_TPI.L_** | GAP.L (ABC) ↔ DHAP.L (ABC) |
| **V_GK.L_** | Glycerol (ABC) → DHAP.L (ABC) |
| **V_GAPDH.L_** | BPG.L (ABC) → GAP.L (ABC) |
| **V_Enol.L_** | PEP.L (ABC) → BPG.L (ABC) |
| **V_PEPCK.L_** | Oac.L (ABCD) → PEP.L (ABC) + CO_2_ (D) |
| **V_PK+ME.L_** | PEP.L (ABC) → Pyr.L (ABC) |
| **V_PC.L_** | Pyr.L (ABC) + CO_2_ (D) → Oac.L (ABCD) |
| **V_Lac.source_** | Lac (ABC) → Lac.L (ABC) |
| **V_LDH.L_** | Lac.L (ABC) ↔ Pyr.L (ABC) |
| **V_CS.L_** | Oac.L (ABCD) + AcCoA.L (EF) → Cit.L (DCBFEA) |
| **V_ICDH.L_** | Cit.L (ABCDEF) ↔ aKG.L (ABCDE) + CO_2_ (F) |
| **V_aKGDH.L_** | aKG.L (ABCDE) → Suc.L (BCDE) + CO_2_ (A) |
| **V_GDH.L_** | Glu.L (ABCDE) ↔ aKG.L (ABCDE) |
| **V_Glu.exchange_** | Gln.L (ABCDE) ↔ Glu.L (ABCDE) |
| **V_SDH.L_** | Suc.L (ABCD) ↔ Fum.L (ABCD) |
| **V_FM.L_** | Fum.L (ABCD) ↔ Mal.L (ABCD) |
| **V_MDH.L_** | Mal.L (ABCD) ↔ Oac.L (ABCD) |
| **V_Fat.source_** | Fat (AB) → AcCoA.L (AB) |
| **V_CO2.source_** | Bicarb.source (A) → CO_2_ (A) |
| **V_CO2.sink_** | CO_2_ (A) → Bicarb.sink (A) |
| **Extrahepatic Compartment** |  |
| **V_Lac.infusion_** | Lac.isotope (ABC) → Lac.P (ABC) |
| **V_Lac.exchange_** | Lac.P (ABC) → Lac.L (ABC) |
| **V_Gln.source_** | Gln (ABCDE) → Gln.P (ABCDE) |
| **V_Gln.exchange_** | Gln.P (ABCDE) ↔ Gln.L (ABCDE) |
| **V_HK.M_** | Glucose (ABCDEF) → F6P.M (ABCDEF) |
| **V_PYGM.M_** | Glycogen (ABCDEF) → F6P.M (ABCDEF) |
| **V_Aldo.M_** | F6P.M (ABCDEF) → GAP.M (CBA) + DHAP.M (DEF) |
| **V_TPI.M_** | DHAP.M (ABC) ↔ GAP.M (ABC) |
| **V_Enol.M_** | GAP.M (ABC) → PEP.M (ABC) |
| **V_PK.M_** | PEP.M (ABC) → Pyr.M (ABC) |
| **V_LDH.M_** | Pyr.M (ABC) ↔ Lac.P (ABC) |
| **V_PyrOx.M_** | Pyr.M (ABC) → CO2 (A) + CO_2_ (B) + CO_2_ (C) |
| **Pseudofluxes representing mixing of the two compartments** | |
| **V_Lac.hot_** | 0*Lac.L (ABC) → Lac.mix (ABC) |
| **V_Lac.cold_** | 0*Lac.cold (ABC) → Lac.mix (ABC) |
| **V_Lac.sink_** | Lac.mix (ABC) → Lac.sink (ABC) |
| **V_Pyr.hot_** | 0*Pyr.L (ABC) → Pyr.mix (ABC) |
| **V_Pyr.cold_** | 0*Pyr.cold (ABC) → Pyr.mix (ABC) |
| **V_Pyr.sink_** | Pyr.mix (ABC) → Pyr.sink (ABC) |

Table S1. List of model reactions.

|  | **WT chow** | | | **WT WD** | | | **WT WD**  **+CDN1163 x4** | | | **KO chow** | | | **KO WD** | | | **KO WD**  **+CDN1163 x24** | | |
| --- | --- | --- | --- | --- | --- | --- | --- | --- | --- | --- | --- | --- | --- | --- | --- | --- | --- | --- |
| **14:0** | 0.11 | ± | 0.04 | 0.20 | ± | 0.01 | 0.18 | ± | 0.03 | 0.00 | ± | 0.00 | 0.00 | ± | 0.00 | 0.10 | ± | 0.06 |
| **16:0** | 23.54 | ± | 0.60 | 19.25 | ± | 0.18 * | 19.38 | ± | 0.22 * | 18.12 | ± | 0.73 | 18.19 | ± | 0.34 | 19.46 | ± | 0.18 |
| **16:1** | 1.16 | ± | 0.27 | 2.41 | ± | 0.12 | 1.55 | ± | 0.15 | 1.12 | ± | 0.17 | 1.66 | ± | 0.05 | 0.99 | ± | 0.05 |
| **18:0** | 16.66 | ± | 0.55 | 14.04 | ± | 0.29 * | 15.54 | ± | 0.35 | 17.76 | ± | 0.82 | 14.56 | ± | 0.20 | 17.73 | ± | 0.41 |
| **18:1ω9** | 8.67 | ± | 1.22 | 12.88 | ± | 0.08 * | 11.21 | ± | 0.55 *# | 9.28 | ± | 1.39 | 14.57 | ± | 0.32 * | 8.94 | ± | 0.25 # |
| **18:1ω7** | 1.55 | ± | 0.35 | 3.67 | ± | 0.19 * | 2.84 | ± | 0.21 | 3.80 | ± | 0.80 | 7.27 | ± | 0.18 * | 3.13 | ± | 0.21 # |
| **18:2** | 14.44 | ± | 1.07 | 10.17 | ± | 0.51 * | 11.33 | ± | 0.59 * | 11.17 | ± | 1.14 | 6.85 | ± | 0.26 * | 10.67 | ± | 0.36 # |
| **18:3ω6** | 0.08 | ± | 0.05 | 0.19 | ± | 0.05 | 0.16 | ± | 0.05 | 0.14 | ± | 0.06 | 0.00 | ± | 0.00 | 0.00 | ± | 0.00 |
| **18:3ω3** | 0.11 | ± | 0.03 | 0.30 | ± | 0.08 | 0.25 | ± | 0.08 | 0.23 | ± | 0.10 | 0.54 | ± | 0.02 | 0.00 | ± | 0.00 |
| **20:3ω6** | 1.69 | ± | 0.26 | 3.61 | ± | 0.21 * | 2.59 | ± | 0.18 | 3.30 | ± | 0.23 | 4.19 | ± | 0.07 | 3.19 | ± | 0.12 |
| **20:4** | 15.39 | ± | 1.25 | 19.84 | ± | 0.52 * | 20.20 | ± | 0.66 * | 17.41 | ± | 0.43 | 19.27 | ± | 0.66 * | 21.14 | ± | 0.16 *# |
| **20:5** | 1.73 | ± | 0.36 | 0.49 | ± | 0.05 | 0.74 | ± | 0.12 | 1.45 | ± | 0.36 | 0.10 | ± | 0.10 | 0.45 | ± | 0.11 |
| **22:4ω6** | 0.00 | ± | 0.00 | 0.37 | ± | 0.04 | 0.17 | ± | 0.06 | 0.00 | ± | 0.00 | 0.10 | ± | 0.10 | 0.00 | ± | 0.00 |
| **22:5ω6** | 0.10 | ± | 0.10 | 0.70 | ± | 0.22 | 0.41 | ± | 0.11 | 0.21 | ± | 0.21 | 0.75 | ± | 0.20 | 0.00 | ± | 0.00 |
| **22:5ω3** | 0.67 | ± | 0.09 | 0.44 | ± | 0.01 | 0.34 | ± | 0.12 | 0.66 | ± | 0.18 | 0.05 | ± | 0.05 | 0.41 | ± | 0.06 |
| **22:6** | 14.11 | ± | 0.90 | 11.44 | ± | 0.28 * | 13.12 | ± | 0.71 # | 15.36 | ± | 1.13 | 11.91 | ± | 0.21 * | 13.79 | ± | 0.23 *# |

Table S2. Hepatic phospholipids fatty acid composition. Data are presented as mean (percent of lipid composition) ± SEM (n ≥ 6). **p* < 0.05 versus chow, #*p* < 0.05 versus WD.

|  | **WT chow** | | | **WT WD** | | | **WT WD**  **+CDN1163 x4** | | | **KO chow** | | | **KO WD** | | | **KO WD**  **+CDN1163 x24** | | |
| --- | --- | --- | --- | --- | --- | --- | --- | --- | --- | --- | --- | --- | --- | --- | --- | --- | --- | --- |
| **14:0** | 0.82 | ± | 0.19 | 1.64 | ± | 0.12 | 1.42 | ± | 0.11 | 0.66 | ± | 0.15 | 0.92 | ± | 0.03 | 0.65 | ± | 0.02 |
| **16:0** | 27.42 | ± | 0.51 | 24.40 | ± | 0.72 | 25.23 | ± | 0.75 | 24.36 | ± | 0.94 | 23.83 | ± | 0.20 | 27.15 | ± | 0.57 *# |
| **16:1** | 3.63 | ± | 0.78 | 8.78 | ± | 0.39 * | 5.16 | ± | 0.51 # | 5.47 | ± | 0.61 | 6.46 | ± | 0.15 | 5.50 | ± | 0.21 |
| **18:0** | 1.97 | ± | 0.15 | 1.36 | ± | 0.05 | 2.13 | ± | 0.18 | 1.47 | ± | 0.09 | 0.97 | ± | 0.07 | 1.56 | ± | 0.08 |
| **18:1ω9** | 32.51 | ± | 4.85 | 51.97 | ± | 1.27 * | 49.91 | ± | 1.43 * | 47.19 | ± | 2.23 | 55.55 | ± | 0.55 * | 49.55 | ± | 0.72 # |
| **18:1ω7** | 2.28 | ± | 1.07 | 5.56 | ± | 0.64 * | 6.14 | ± | 0.38 * | 5.73 | ± | 0.82 | 9.35 | ± | 0.42 * | 5.46 | ± | 0.28 # |
| **18:2** | 20.65 | ± | 3.93 | 4.67 | ± | 0.23 * | 7.74 | ± | 1.57 *# | 9.63 | ± | 2.01 | 2.37 | ± | 0.09 * | 8.53 | ± | 0.61 # |
| **18:3ω6** | 0.37 | ± | 0.10 | 0.12 | ± | 0.09 | 0.06 | ± | 0.06 | 0.08 | ± | 0.03 | 0.00 | ± | 0.00 | 0.00 | ± | 0.00 |
| **18:3ω3** | 0.98 | ± | 0.25 | 0.00 | ± | 0.00 | 0.00 | ± | 0.00 | 0.00 | ± | 0.00 | 0.00 | ± | 0.00 | 0.22 | ± | 0.05 |
| **20:3ω6** | 0.40 | ± | 0.11 | 0.29 | ± | 0.04 | 0.18 | ± | 0.09 | 0.39 | ± | 0.10 | 0.13 | ± | 0.01 | 0.28 | ± | 0.06 |
| **20:4** | 0.92 | ± | 0.23 | 0.40 | ± | 0.04 | 0.36 | ± | 0.17 | 0.42 | ± | 0.11 | 0.11 | ± | 0.01 | 0.40 | ± | 0.07 |
| **20:5** | 1.31 | ± | 0.34 | 0.10 | ± | 0.07 | 0.12 | ± | 0.12 | 0.61 | ± | 0.19 | 0.13 | ± | 0.02 | 0.00 | ± | 0.00 |
| **22:4ω6** | 0.00 | ± | 0.00 | 0.06 | ± | 0.04 | 0.00 | ± | 0.00 | 0.05 | ± | 0.03 | 0.02 | ± | 0.02 | 0.00 | ± | 0.00 |
| **22:5ω6** | 1.13 | ± | 0.30 | 0.10 | ± | 0.04 | 0.24 | ± | 0.15 | 0.93 | ± | 0.27 | 0.00 | ± | 0.00 | 0.05 | ± | 0.03 |
| **22:5ω3** | 5.61 | ± | 1.40 | 0.47 | ± | 0.05 | 1.31 | ± | 0.65 | 3.01 | ± | 0.91 | 0.15 | ± | 0.03 | 0.66 | ± | 0.10 |
| **22:6** | 0.82 | ± | 0.19 | 1.64 | ± | 0.12 * | 1.42 | ± | 0.11 * | 0.66 | ± | 0.15 | 0.92 | ± | 0.03 | 0.65 | ± | 0.02 |

Table S3. Hepatic triglycerides fatty acid composition. Data are presented as mean (percent of lipid composition) ± SEM (n ≥ 6). **p* < 0.05 versus chow, #*p* < 0.05 versus WD.

|  | **WT chow** | | | **WT WD** | | | **WT WD**  **+CDN1163 x4** | | | **KO chow** | | | **KO WD** | | | **KO WD**  **+CDN1163 x24** | | |
| --- | --- | --- | --- | --- | --- | --- | --- | --- | --- | --- | --- | --- | --- | --- | --- | --- | --- | --- |
| **14:0** | 0.45 | ± | 0.21 | 1.35 | ± | 0.14 | 1.33 | ± | 0.15 | 1.08 | ± | 0.21 | 0.98 | ± | 0.14 | 0.44 | ± | 0.10 |
| **16:0** | 23.66 | ± | 0.80 | 28.73 | ± | 1.72 | 23.98 | ± | 0.99 | 32.15 | ± | 1.94 | 29.82 | ± | 1.94 | 22.62 | ± | 0.53 *# |
| **16:1** | 2.07 | ± | 0.98 | 5.31 | ± | 0.64 | 2.91 | ± | 0.52 | 3.63 | ± | 0.49 | 5.02 | ± | 0.26 | 4.57 | ± | 0.20 |
| **18:0** | 16.23 | ± | 5.18 | 13.39 | ± | 4.26 | 17.79 | ± | 2.01 | 23.91 | ± | 4.91 | 8.52 | ± | 1.98 * | 6.40 | ± | 0.60 * |
| **18:1ω9** | 25.89 | ± | 5.09 | 36.77 | ± | 4.46 * | 31.83 | ± | 3.94 * | 27.04 | ± | 5.39 | 39.61 | ± | 2.96 * | 44.20 | ± | 0.97 * |
| **18:1ω7** | 2.04 | ± | 0.50 | 4.89 | ± | 0.42 | 5.38 | ± | 0.50 | 4.95 | ± | 0.71 | 10.55 | ± | 0.77 * | 4.00 | ± | 0.18 # |
| **18:2** | 13.99 | ± | 3.57 | 6.25 | ± | 2.77 * | 13.13 | ± | 2.10 # | 4.25 | ± | 0.87 | 1.91 | ± | 0.42 * | 11.01 | ± | 0.74 *# |
| **18:3ω6** | 0.10 | ± | 0.10 | 0.00 | ± | 0.00 | 0.00 | ± | 0.00 | 0.00 | ± | 0.00 | 0.00 | ± | 0.00 | 0.00 | ± | 0.00 |
| **18:3ω3** | 0.17 | ± | 0.17 | 0.00 | ± | 0.00 | 0.00 | ± | 0.00 | 0.00 | ± | 0.00 | 0.90 | ± | 0.56 | 0.00 | ± | 0.00 |
| **20:3ω6** | 0.56 | ± | 0.24 | 0.00 | ± | 0.00 | 0.00 | ± | 0.00 | 0.00 | ± | 0.00 | 0.00 | ± | 0.00 | 0.27 | ± | 0.13 |
| **20:4** | 5.30 | ± | 0.55 | 2.27 | ± | 0.28 | 2.58 | ± | 0.33 | 1.40 | ± | 1.12 | 0.98 | ± | 0.62 | 2.93 | ± | 0.30 |
| **20:5** | 3.40 | ± | 1.16 | 0.00 | ± | 0.00 | 0.00 | ± | 0.00 | 0.00 | ± | 0.00 | 0.00 | ± | 0.00 | 0.00 | ± | 0.00 |
| **22:4ω6** | 0.00 | ± | 0.00 | 0.00 | ± | 0.00 | 0.00 | ± | 0.00 | 0.00 | ± | 0.00 | 0.00 | ± | 0.00 | 0.00 | ± | 0.00 |
| **22:5ω6** | 0.00 | ± | 0.00 | 0.00 | ± | 0.00 | 0.00 | ± | 0.00 | 0.00 | ± | 0.00 | 0.00 | ± | 0.00 | 0.00 | ± | 0.00 |
| **22:5ω3** | 0.23 | ± | 0.23 | 0.00 | ± | 0.00 | 0.00 | ± | 0.00 | 0.00 | ± | 0.00 | 0.00 | ± | 0.00 | 0.00 | ± | 0.00 |
| **22:6** | 5.92 | ± | 0.99 | 1.03 | ± | 0.43 | 1.07 | ± | 0.48 | 1.58 | ± | 1.09 | 1.71 | ± | 1.26 | 3.55 | ± | 0.15 |

Table S4. Hepatic diacylglycerides fatty acid composition. Data are presented as mean (percent of lipid composition) ± SEM (n ≥ 6). **p* < 0.05 versus chow, #*p* < 0.05 versus WD.

|  | **WT chow** | | | **WT WD** | | | **WT WD**  **+CDN1163 x4** | | | **KO chow** | | | **KO WD** | | | **KO WD**  **+CDN1163 x24** | | |
| --- | --- | --- | --- | --- | --- | --- | --- | --- | --- | --- | --- | --- | --- | --- | --- | --- | --- | --- |
| **14:0** | 0.64 | ± | 0.08 | 1.12 | ± | 0.08 | 1.06 | ± | 0.15 | 0.34 | ± | 0.14 | 0.71 | ± | 0.01 | 0.65 | ± | 0.03 |
| **16:0** | 33.30 | ± | 0.56 | 28.07 | ± | 0.25 * | 30.79 | ± | 0.68 *# | 32.06 | ± | 1.02 | 28.65 | ± | 0.70 * | 29.90 | ± | 0.82 |
| **16:1** | 2.55 | ± | 0.67 | 7.29 | ± | 0.30 * | 4.43 | ± | 0.54 # | 4.10 | ± | 0.22 | 5.12 | ± | 0.14 | 4.63 | ± | 0.19 |
| **18:0** | 11.57 | ± | 1.35 | 9.34 | ± | 0.48 | 11.48 | ± | 0.51 | 14.33 | ± | 1.00 | 12.63 | ± | 1.52 | 10.26 | ± | 0.70 *# |
| **18:1ω9** | 18.47 | ± | 2.22 | 33.16 | ± | 1.07 * | 28.94 | ± | 1.45 *# | 25.90 | ± | 1.53 | 32.79 | ± | 1.22 * | 31.00 | ± | 0.77 * |
| **18:1ω7** | 1.65 | ± | 0.48 | 4.30 | ± | 0.31 * | 3.71 | ± | 0.36 * | 5.27 | ± | 0.49 | 9.34 | ± | 0.32 * | 4.91 | ± | 0.32 # |
| **18:2** | 15.03 | ± | 2.30 | 7.31 | ± | 0.45 * | 8.72 | ± | 1.21 * | 9.72 | ± | 1.79 | 3.65 | ± | 0.16 * | 9.22 | ± | 0.46 # |
| **18:3ω6** | 0.14 | ± | 0.09 | 0.12 | ± | 0.12 | 0.00 | ± | 0.00 | 0.00 | ± | 0.00 | 0.00 | ± | 0.00 | 0.00 | ± | 0.00 |
| **18:3ω3** | 0.76 | ± | 0.20 | 0.00 | ± | 0.00 | 0.15 | ± | 0.10 | 0.00 | ± | 0.00 | 0.39 | ± | 0.24 | 0.06 | ± | 0.06 |
| **20:3ω6** | 0.65 | ± | 0.05 | 0.97 | ± | 0.05 | 0.67 | ± | 0.14 | 0.00 | ± | 0.00 | 0.43 | ± | 0.26 | 0.88 | ± | 0.05 |
| **20:4** | 5.04 | ± | 0.22 | 5.70 | ± | 0.33 | 6.06 | ± | 0.36 | 4.06 | ± | 0.31 | 5.44 | ± | 0.56 | 5.25 | ± | 0.33 |
| **20:5** | 2.26 | ± | 0.85 | 0.26 | ± | 0.08 | 0.51 | ± | 0.23 | 0.47 | ± | 0.47 | 0.00 | ± | 0.00 | 0.17 | ± | 0.12 |
| **22:4ω6** | 0.00 | ± | 0.00 | 0.09 | ± | 0.09 | 0.00 | ± | 0.00 | 0.00 | ± | 0.00 | 0.00 | ± | 0.00 | 0.00 | ± | 0.00 |
| **22:5ω6** | 0.00 | ± | 0.00 | 0.08 | ± | 0.08 | 0.00 | ± | 0.00 | 0.00 | ± | 0.00 | 0.00 | ± | 0.00 | 0.00 | ± | 0.00 |
| **22:5ω3** | 0.97 | ± | 0.25 | 0.04 | ± | 0.04 | 0.20 | ± | 0.10 | 0.00 | ± | 0.00 | 0.00 | ± | 0.00 | 0.00 | ± | 0.00 |
| **22:6** | 6.97 | ± | 1.28 | 2.17 | ± | 0.12 * | 3.28 | ± | 0.63 * | 3.77 | ± | 0.57 | 0.86 | ± | 0.52 * | 3.07 | ± | 0.20 # |

Table S5. Hepatic free fatty acids composition. Data are presented as mean (percent of lipid composition) ± SEM (n ≥ 6). **p* < 0.05 versus chow, #*p* < 0.05 versus WD.

|  | **WT chow** | | | **WT WD** | | | **WT WD**  **+CDN1163 x4** | | | **KO chow** | | | **KO WD** | | | **KO WD**  **+CDN1163 x24** | | |
| --- | --- | --- | --- | --- | --- | --- | --- | --- | --- | --- | --- | --- | --- | --- | --- | --- | --- | --- |
| **14:0** | 2.08 | ± | 1.41 | 0.00 | ± | 0.00 | 0.71 | ± | 0.47 | 0.27 | ± | 0.27 | 0.00 | ± | 0.00 | 1.48 | ± | 0.09 |
| **16:0** | 41.07 | ± | 2.33 | 45.41 | ± | 0.89 | 45.98 | ± | 4.91 | 42.71 | ± | 2.71 | 46.71 | ± | 2.47 | 33.56 | ± | 1.88 *# |
| **16:1** | 0.00 | ± | 0.00 | 0.00 | ± | 0.00 | 0.00 | ± | 0.00 | 0.00 | ± | 0.00 | 0.00 | ± | 0.00 | 0.00 | ± | 0.00 |
| **18:0** | 24.75 | ± | 3.29 | 22.73 | ± | 3.95 | 18.50 | ± | 1.66 * | 21.79 | ± | 1.13 | 23.35 | ± | 1.40 | 22.83 | ± | 1.41 |
| **18:1ω9** | 17.19 | ± | 2.12 | 18.24 | ± | 1.62 | 20.33 | ± | 0.60 | 24.07 | ± | 2.65 | 19.43 | ± | 2.17 | 12.78 | ± | 0.83 *# |
| **18:1ω7** | 6.74 | ± | 1.92 | 10.17 | ± | 1.58 | 7.06 | ± | 1.64 | 8.53 | ± | 1.12 | 7.41 | ± | 0.56 | 1.20 | ± | 0.21 *# |
| **18:2** | 0.00 | ± | 0.00 | 0.00 | ± | 0.00 | 1.73 | ± | 1.10 | 1.06 | ± | 1.06 | 0.00 | ± | 0.00 | 3.45 | ± | 0.34 |
| **18:3ω6** | 0.00 | ± | 0.00 | 0.00 | ± | 0.00 | 0.00 | ± | 0.00 | 0.00 | ± | 0.00 | 0.00 | ± | 0.00 | 0.00 | ± | 0.00 |
| **18:3ω3** | 0.00 | ± | 0.00 | 0.00 | ± | 0.00 | 0.00 | ± | 0.00 | 0.00 | ± | 0.00 | 0.00 | ± | 0.00 | 0.00 | ± | 0.00 |
| **20:3ω6** | 0.00 | ± | 0.00 | 0.00 | ± | 0.00 | 0.00 | ± | 0.00 | 0.00 | ± | 0.00 | 0.00 | ± | 0.00 | 0.00 | ± | 0.00 |
| **20:4** | 4.87 | ± | 2.99 | 0.00 | ± | 0.00 | 0.00 | ± | 0.00 | 0.00 | ± | 0.00 | 0.00 | ± | 0.00 | 0.00 | ± | 0.00 |
| **20:5** | 0.00 | ± | 0.00 | 0.00 | ± | 0.00 | 3.38 | ± | 2.16 | 0.00 | ± | 0.00 | 0.00 | ± | 0.00 | 12.98 | ± | 2.04 *# |
| **22:4ω6** | 0.00 | ± | 0.00 | 0.00 | ± | 0.00 | 0.00 | ± | 0.00 | 0.00 | ± | 0.00 | 0.00 | ± | 0.00 | 0.00 | ± | 0.00 |
| **22:5ω6** | 3.30 | ± | 1.35 | 3.44 | ± | 2.11 | 0.00 | ± | 0.00 | 1.56 | ± | 1.56 | 3.10 | ± | 1.96 | 0.00 | ± | 0.00 |
| **22:5ω3** | 0.00 | ± | 0.00 | 0.00 | ± | 0.00 | 0.00 | ± | 0.00 | 0.00 | ± | 0.00 | 0.00 | ± | 0.00 | 0.00 | ± | 0.00 |
| **22:6** | 0.00 | ± | 0.00 | 0.00 | ± | 0.00 | 2.32 | ± | 2.32 | 0.00 | ± | 0.00 | 0.00 | ± | 0.00 | 11.72 | ± | 2.47 *# |

Table S6. Hepatic ceramides fatty acid composition. Data are presented as mean (percent of lipid composition) ± SEM (n ≥ 6). **p* < 0.05 versus chow, #*p* < 0.05 versus WD.

**SUPPLEMENTAL REFERENCES**

1. Antoniewicz, M. R., J. K. Kelleher, and G. Stephanopoulos. 2006. Determination of confidence intervals of metabolic fluxes estimated from stable isotope measurements. *Metab Eng*. **8**: 324-337.

2. Steele, R., J. S. Wall, de Bodo R C, and N. Altszuler. 1956. Measurement of Size and Turnover Rate of Body Glucose Pool by the Isotope Dilution Method. *American Journal of Physiology*. **187**: 15–24.

3. Bankhead, P., M. B. Loughrey, J. A. Fernández, Y. Dombrowski, D. G. McArt, P. D. Dunne, S. McQuaid, R. T. Gray, L. J. Murray, H. G. Coleman, J. A. James, M. Salto-Tellez, and P. W. Hamilton. 2017. QuPath: Open source software for digital pathology image analysis. *Sci Rep*. **7**: 1–7.

4. Hasenour, C. M., M. Rahim, and J. D. Young. 2020. In Vivo Estimates of Liver Metabolic Flux Assessed by 13C-Propionate and 13C-Lactate Are Impacted by Tracer Recycling and Equilibrium Assumptions. *Cell Rep*. **32:** 107986.

5. Veech, R. L., L. V. Eggleston, and H. A. Krebs. 1969. The redox state of free nicotinamide-adenine dinucleotide phosphate in the cytoplasm of rat liver. *Biochem J*. **115**: 609–619.

6. Fan, X., H. Yao, X. Liu, Q. Shi, L. Lv, P. Li, R. Wang, T. Tang, and K. Qi. 2020. High-Fat Diet Alters the Expression of Reference Genes in Male Mice. *Front Nutr*. **7**: 1–14.

7. Tomfohr, J., J. Lu, and T. B. Kepler. 2005. Pathway level analysis of gene expression using singular value decomposition. *BMC Bioinformatics*. **6**: 1–11.
